# Finite-Temperature Evolution of Frenkel Defects in Hybrid Perovskites: Healing and Lead-Methylammonium Antisite Pairs

**DOI:** 10.1021/acsami.6c02743

**Published:** 2026-04-09

**Authors:** Jgor Pensè Schone, Simone Argiolas, Alessio Gagliardi, Gohar Ali Siddiqui, Alessio Filippetti, Alessandro Mattoni

**Affiliations:** † CNR - Istituto Officina dei Materiali (IOM) Cagliari, Cittadella Universitaria, Monserrato, (CA) 09042, Italy; ‡ Dipartimento di Fisica, Università degli Studi di Cagliari, Cittadella Universitaria, Monserrato, (CA) 09042, Italy; § Chair of Simulation of Nanosystems for Energy Conversion, Department of Electrical Engineering, TUM School of Computation, Information and Technology, Atomistic Modeling Center (AMC), Munich Data Science Institute (MDSI), 9184 Technical University of Munich, Garching 85748, Germany

**Keywords:** MD, point defects, self-healing, metadynamics

## Abstract

Hybrid halide perovskites exhibit remarkable defect tolerance, yet the microscopic origin of this resilience and its limits remain debated. In this work, we employ a combined approach of finite-temperature molecular dynamics and enhanced-sampling metadynamics to investigate the atomistic formation and evolution of Frenkel defects in the prototypical MAPbI_3_ lattice. By inducing local perturbations in the stoichiometric crystal, we reconstruct the free-energy profiles and mechanistic pathways for the formation and evolution of defects for all three constituent species. Our results reveal a fundamental difference in the material’s defect physics. For the monovalent species (iodine and methylammonium), the soft lattice facilitates rapid self-healing via concerted exchange and direct recombination, effectively suppressing the accumulation of isolated defects. Conversely, for the lead sublattice, the initial perturbation triggers an irreversible structural relaxation into a stable double antisite complex (*Pb*_MA_ + *MA*_Pb_), which acts as a deep thermodynamic trap. Large-scale simulations confirm these findings, demonstrating that mobile monovalent defects have a larger interaction range and tend to spontaneously recombine due to short-range instability; while the less mobile lead-based antisites persist as the most energetically favorable separated defect state. These findings provide a mechanistic rationale for the intrinsic self-healing capability of the hybrid framework while identifying the pairs of lead-molecule antisites as the critical bottleneck for long-term electronic stability.

## Graphical abstract

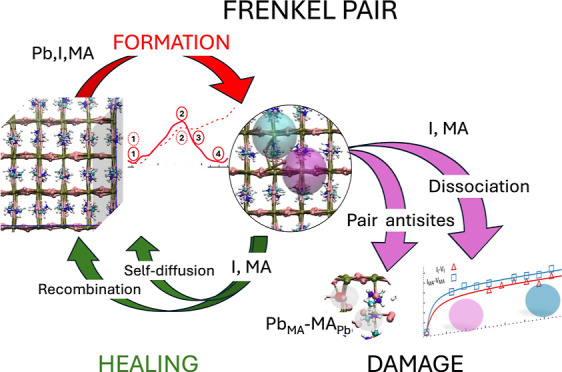

## Introduction

Hybrid organic–inorganic perovskites have emerged as an important class of semiconductors, impacting the field of modern optoelectronics.^1,2^ Characterized by a distinct crystal structure and exceptional compositional flexibility, these materials offer unprecedented tunability of their optical and electronic properties.^3–5^ This versatility provides access to a diverse range of applications, ranging from high-performance light-emitting diodes (LEDs), chiro-optics, and tandem solar cells to spintronics and neuromorphic computing.^6–11^

Among these, methylammonium lead iodide (MAPbI_3_ or MAPI) is the archetypal system in the photovoltaic field.^12,13^ Since its debut, the power conversion efficiency (PCE) of perovskite solar cells has skyrocketed from 3.8% in 2009 ^14^ to certified values now exceeding 26%,^15^ effectively rivaling crystalline silicon technologies. The success of MAPbI_3_ and its derivatives is linked to their unique processing advantages. Unlike traditional semiconductors such as silicon or GaAs, hybrid perovskites can be fabricated using low-cost solution-based methods (e.g., spin coating,^16,17^ spray coating^18^) at low temperatures, which inevitably results in a high density of defects.^19,20^ This apparent paradox of high efficiency in a defect-rich material was initially rationalized by the peculiar electronic band structure of lead halide perovskites, leading to the concept of defect tolerance. The valence band maximum (VBM) in MAPI originates from the antibonding interaction between I­(5*p*) and Pb­(6*s*) orbitals, while the conduction band minimum (CBM) is mainly derived from Pb (6*p*) and I (6*s*) states, strongly affected by relativistic spin–orbit coupling (SOC).^21^ As early theoretical studies suggested, this electronic structure, characterized by small effective masses and large electronic dielectric screening,^22^ gives rise to highly dispersive band edges, such that many of the energetically favorable native point defects introduce only shallow states close to the band edges and, therefore, do not act as efficient nonradiative recombination centers.^23–26^

However, recent investigations have emphasized the necessity of defect passivation and density control, as deep-level defects within the band gap are now recognized to limit device performance.^27–31^ For instance, Ni et al. mapped the defect evolution in operating devices, revealing that negatively charged iodine interstitials *I*_i_^–^ accumulate at the interfaces under illumination and reverse bias. They demonstrated that while *I*_i_^+^ may be less detrimental, the *I*_i_^–^ species acts as a persistent deep trap that drives performance degradation.^32^ Complementing this, Meggiolaro et al. identified *I*_*i*_ as a source of deep traps capturing both carriers; yet, they also elucidated a chemical passivation mechanism driven by iodine redox chemistry, where the harmful hole-trapping state (*I*_i_^–^) can be converted into the kinetically inactive cation (*I*_i_^+^) under mild oxidizing conditions.^33^ Nevertheless, the defect landscape extends beyond simple vacancies or interstitials. Cation–anion misplacements (antisites) and defect complexes have been identified as potentially detrimental centers.^28,34,35^ In particular, computational studies on related hybrid perovskite systems (e.g., FAPbI_3_) indicate that Frenkel pairs or antisite complexes can act as stable deep traps, contrasting with the shallow nature of their isolated constituents.^36^

The influence of defects on the properties of hybrid perovskites is not determined solely by the static electronic nature of point defects, but rather arises from a complex set of processes encompassing their formation, interaction, migration, and redistribution.

The structural softness of the lattice facilitates significant ion migration, unlocking a self-healing capability that promotes the annihilation of defects.^37,38^ This implies that the impact of a defect is determined not only by its static formation energy but also by its kinetic evolution and lifetime within the soft lattice. Such dynamic self-healing mechanisms have recently been observed in all-inorganic metal halide perovskites: in CsPbBr_3_, Miskin et al. demonstrated that double antisite defects can annihilate through a low-barrier concerted exchange mechanism.^39^ Similarly, Lv et al. demonstrated that in CsPbI_3_ the cooperative dynamics of iodide defects enable long-range annihilation, demonstrating that interstitial–vacancy pairs preferentially recombine rather than dissociate, thereby underpinning a robust self-healing mechanism.^40^

However, capturing the entire kinetic evolution of such complex defects, from their spontaneous formation to their ultimate fate, remains a significant challenge. As summarized in Table 1, the current literature on MAPI presents a scattered landscape of formation energies, largely derived from static calculations in small simulation cells, where finite-size effects and periodic–image interactions hinder the reliable treatment of multiple point defects. By neglecting finite-temperature fluctuations and lattice dynamics, these methods offer limited insight into the competing pathways that determine whether a nascent defect complex will successfully heal or stabilize into a detrimental trap.

**Table 1:** Formation energies of Frenkel pairs in MAPbI_3_ from selected theoretical studies^a^

formation energy (eV)			
iodine (I)	MA	lead (Pb)	method
0.86–1.36	–	–	DFT (PBE/HSE-SOC)^49^
–	0.07^b^	2.15^b^	DFT (PBEsol + SOC)^50^
0.13^b^	–	1.13^b^	DFT (PBEsol)^36^
1.26^b^	–	–	DPMD + NEB^40^
0.45–0.85	0.94–1.16	–	AIMD (300 K)^37^
1.12–1.16	–	–	DFT (PBE/HSE + SOC)^51^
0.55–0.86	1.32–1.56	–	DFT (SR-PBE/SOC-PBE)^52^
0.75–1.46	–	–	DFT (PBE)^53^
0.27–0.82	–	–	DFT (PBE + USPP)^54^

a Values are in eV.

b Data calculated for non-MAPbI_3_ perovskite compositions.

In this work, we investigate the physics of point defect formation and evolution within the stoichiometric MAPbI_3_ crystal at finite temperature. By combining large-scale molecular dynamics, based on the MYP0 force-field^41^ and enhanced-sampling metadynamics,^42,43^ we simulate the formation of Frenkel pairs (i.e., pairs formed by an atomic interstitial and its vacancy) for the three constituent species I, CH_3_NH_3_ (MA), and Pb. The MYP0 interatomic potential is well-established for the study of defects-related properties of hybrid perovskites^44–48^ (see Section S1 in the Supporting Information for additional information). This approach allows us to reconstruct the free-energy surface (FES) and track the atomistic evolution of the defects, from the initial formation of bound pairs to either their dissociation at large distances or their recombination and self-healing of the crystal lattice.

Our analysis reveals two contrasting pathways. For the iodine and methylammonium species, the Frenkel pair has a strong propensity for fast self-healing via a dual mechanism of concerted exchange and self-diffusion driven by ionic interactions. Conversely, the lead sublattice follows a divergent trajectory: the local perturbation triggers a cation exchange that relaxes irreversibly into a persistent double antisite complex. Furthermore, by evaluating long-range interaction profiles and the dissociation of the bound pairs at large distances, we highlight the dominant role of electrostatic forces in the Frenkel pair binding and self-healing phenomena. Collectively, these findings provide a mechanistic rationale for the intrinsic resilience of hybrid perovskites while identifying the lead-based double antisite as a primary candidate for long-lived deep trap states.

## Results and Discussion

### Iodine and Methylammonium: Self-Healing through Self-Diffusion

We first examine the formation of Frenkel pairs involving a monovalent ion (i.e., iodide anion or methylammonium cation).

Figure 1 (top panel) displays the Free Energy Surface (FES) for the displacement of a lattice iodine anion as a function of the distance (solid red line). The profile reveals a formation barrier of approximately 1.3 eV, corresponding to the energy required to displace the biased iodine anion from its equilibrium lattice site at the octahedral vertex into an interstitial position, thereby leaving a vacancy behind. Crucially, once this barrier is crossed, the free energy does not plateau into a stable high-energy defect state. Instead, the system relaxes into a new basin thermodynamically indistinguishable from the initial state, albeit atomistically distinct due to the exchange of ions. This energetic feature corresponds to the successful healing of the lattice. The atomistic nature of this evolution is illustrated in Figure 1 (bottom panel). The initial displacement creates a transient bound Frenkel pair, characterized by a separation of ≈3 Å, which we define as the bound Frenkel pair radius, *R*_B_ (snapshot 2).

**Figure 1:**
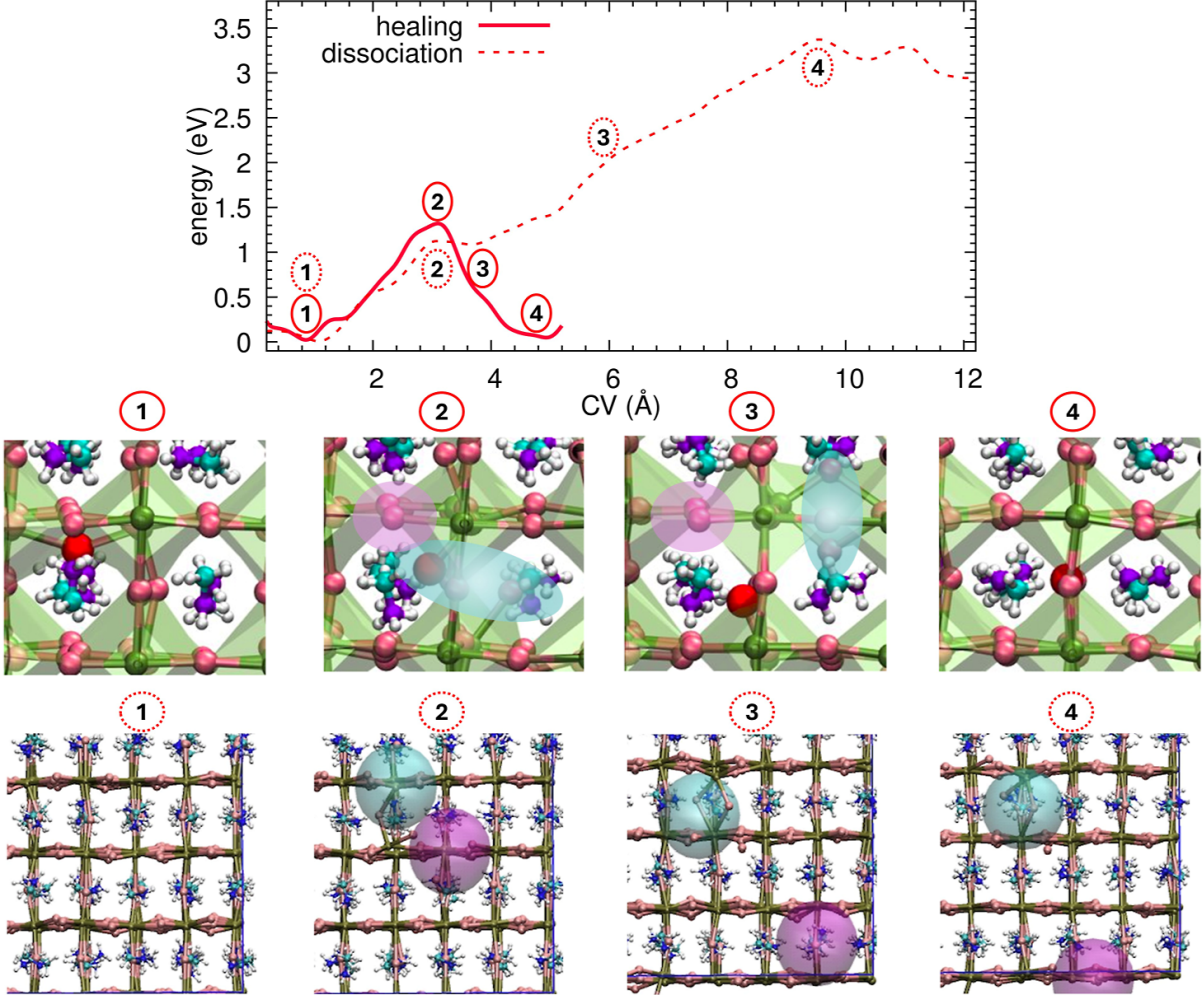
Energetics and dynamics of Iodine Frenkel pairs. Top panel: free-energy profile (FES) for the formation of the Iodine Frenkel pair at 300 K (solid line). The dashed line represents the energy profile along the dissociation pathway. Bottom panel: atomistic comparison of the two competing mechanisms. The labels (1–4) correspond to the energetic states marked in the top panel, distinguished by solid circles (healing) and dashed circles (dissociation): (1) pristine crystal structure; (2) formation of the transient Frenkel pair (common initial state); (3) bifurcation of the pathway: the top row shows a substitution mechanisms involving the reorientation of the FP (healing), while the bottom row shows the increasing separation of defects (dissociation); (4) final state: restoration of the pristine lattice via self-diffusion (top) vs formation of separated, noninteracting defects (bottom). Color code: biased-iodine (red), lattice I (brown), Pb (green), MA [H (white), C (light blue), N (dark blue)].

However, our dynamical simulations reveal that the Frenkel defect, rather than remaining structurally trapped or dissociating into separate vacancies and interstitials, triggers a concerted two-step mechanism. First, the defect undergoes a substitution process where the interstitial biased-iodine displaces a neighboring lattice iodine, effectively reorienting the Frenkel pair (as depicted in snapshot 3). Subsequently, the new interstitial moves toward the vacancy and annihilates with it, relaxing the system back to the pristine crystalline order (Snapshot 4), recovering the initial energy value.

Therefore, this mechanism results in a net exchange of iodine atoms, directly contributing to halide self-diffusion. This finding identifies the local Coulombic interactions as the fundamental driving force that prevents the stabilization of bound Frenkel pairs, favoring instead a rapid self-healing via exchange.

We also explored the alternative evolutionary pathway: the dissociation of the Frenkel pair into isolated, noninteracting defects. Since the dominant tendency for iodine exchange hinders the efficient sampling of long-range separation via metadynamics, we estimated the dissociation cost by analyzing the reverse process: the spontaneous recombination of the defects. We performed a standard molecular dynamics simulation starting from a configuration with the interstitial and vacancy separated by a large distance, following the protocol detailed in the Methods section. By mapping the total energy of the system along this recombination trajectory (dashed line in Figure 1, top panel), we reconstructed the energetic profile for the dissociation.

The profile reveals an energetic penalty, requiring an additional ∼2 eV to separate the vacancy (*V*_I_) and the interstitial (*I*_i_) from the bound state (∼3 Å) to a distance of 15 Å (see dissociation snapshots in the bottom row of Figure 1). This increase in energy, which eventually reaches a plateau, is consistent with the long-range Coulombic attraction between the oppositely charged defects (see long-range Section).

This result demonstrates that, while a small, tightly bound Frenkel pair formed by a local energetic perturbation can, in principle, dissociate into a vacancy and an interstitial, this process is energetically disfavored compared to the system’s strong tendency to restore the pristine crystal—either through direct recombination or via a self-diffusive exchange mechanism, both of which are driven by the high mobility of iodine.

This mechanism confirms the intrinsic self-healing capability of the material, where the lattice spontaneously repairs local damage; a finding that aligns well with experimental observations of dark recovery.^37^ Consequently, this implies that the dissociation of iodine bound Frenkel complexes is not an efficient source of separated interstitials and vacancies.

A qualitatively similar self-healing scenario is observed for the methylammonium cation (*MA* ^+^), although with distinct mechanistic features arising from its molecular nature. Figure 2 (top panel, solid line) presents the FES for the displacement of an MA molecule. The formation of the Frenkel pair (*V*_MA_ + *MA*_i_) requires overcoming a barrier of approximately 2 eV. Our analysis reveals that the transit of the molecular cation into an occupied cage involves a transient freezing of its rotational degrees of freedom to navigate the steric bottleneck. However, this restriction is short-lived. Upon sharing the cage with a second molecule, the rotational dynamics are reactivated by the onset of strong dipole–dipole coupling between the two organic cations. This electrostatic interaction stabilizes the defect configuration, resulting in an energy drop of ∼0.8 eV, which is visible in the FES and is consistent with predictions for MA rotational dynamics using the MYP0 force field.^44^

**Figure 2:**
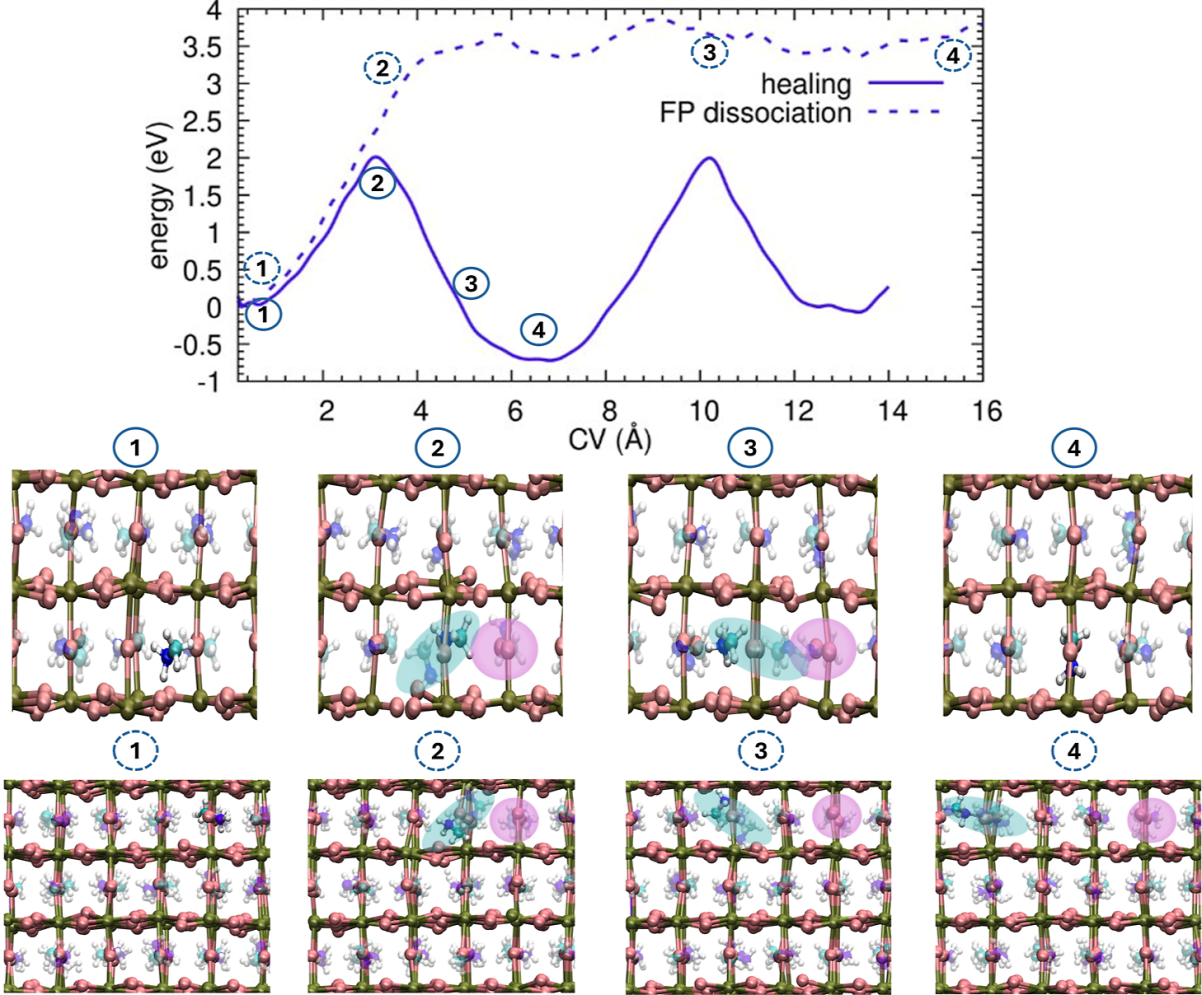
Dynamics of Methylammonium Frenkel pairs. Top panel: free energy surface (FES) for MA Frenkel formation (solid blue line) and dissociation (dashed blue line). Note the energy drop of ∼0.8 eV due to dipole–dipole coupling. Bottom panel: Atomistic comparison of the two competing mechanisms. The labels (1–4) correspond to the energetic states marked in the top panel, distinguished by solid circles (healing) and dashed circles (dissociation): (1) Pristine lattice; (2) formation of MA Frenkel pair; (3) bifurcation of the pathway: the top row shows the substitution mechanism with rotation of Frenkel pair; while the bottom shows the interstitial diffusion toward separation (dissociation); (4) top row: restoration of perfect crystal; bottom row: noninteracting defects. Color code: biased MA (colored), lattice MA (transparent), I (brown), Pb (green).

This intermolecular coupling is the trigger for the restoration mechanism. As illustrated in the atomistic snapshots (Figure 2, bottom panel), the interaction induces the same double mechanisms seen for iodine: the biased molecule assumes a substitutional position (snapshot 3), displacing the resident molecule into an interstitial site. This effectively shifts the Frenkel pair center and leads rapidly to the final annihilation (snapshot 4), restoring the pristine lattice.

We further characterized the dissociation pathway using metadynamics (dashed blue line in Figure 2, top panel). Unlike the iodine case, here we employed repulsive lower walls to inhibit the competing exchange mechanism (see Methods for details). Although molecular mobility is lower than that of iodine, the tendency to swap remains the kinetically favored path, hindering the sampling of direct dissociation without such constraints. The reconstructed FES reveals that dissociation starting from the bound state *R*_B_ ≈ 3.3 Å entails an additional barrier of ∼1.5 eV, resulting in a total energy cost of ∼3.5 eV. Beyond the first unit cell separation (∼6.4 Å), the MA vacancy and the interstitial are almost unbound; however, full dissociation is achieved at larger distances ∼1.4 nm (see below).

This result leads to a dual conclusion: on the one hand, the high energetic cost implies that locally formed Frenkel pairs are thermodynamically prone to heal, behaving as a tightly bound, correlated unit. On the other hand, the energy landscape at larger separations suggests that, once most of the energetic penalty of dissociation is paid, the isolated *MA* ^+^ interstitial can diffuse over free energy barriers of the order of 0.5–0.8 eV through the lattice, likely assisted by the same rotational dynamics that facilitate local exchange.

### Lead: Metastable Antisite Defect Pairs

The lead sublattice exhibits distinct behavior compared to the self-healing mechanisms observed for the monovalent species. Figure 3 (top panel) displays the FES as a function of the divalent Pb displacement. The creation of the initial Frenkel pair requires crossing a significant energy barrier of ∼2.1 eV. This process generates a bound Frenkel pair (*V*_Pb_ + *Pb*_*i*_) characterized by a short separation of *R*_B_ ≈ 2.5 Å (Snapshot 2).

**Figure 3:**
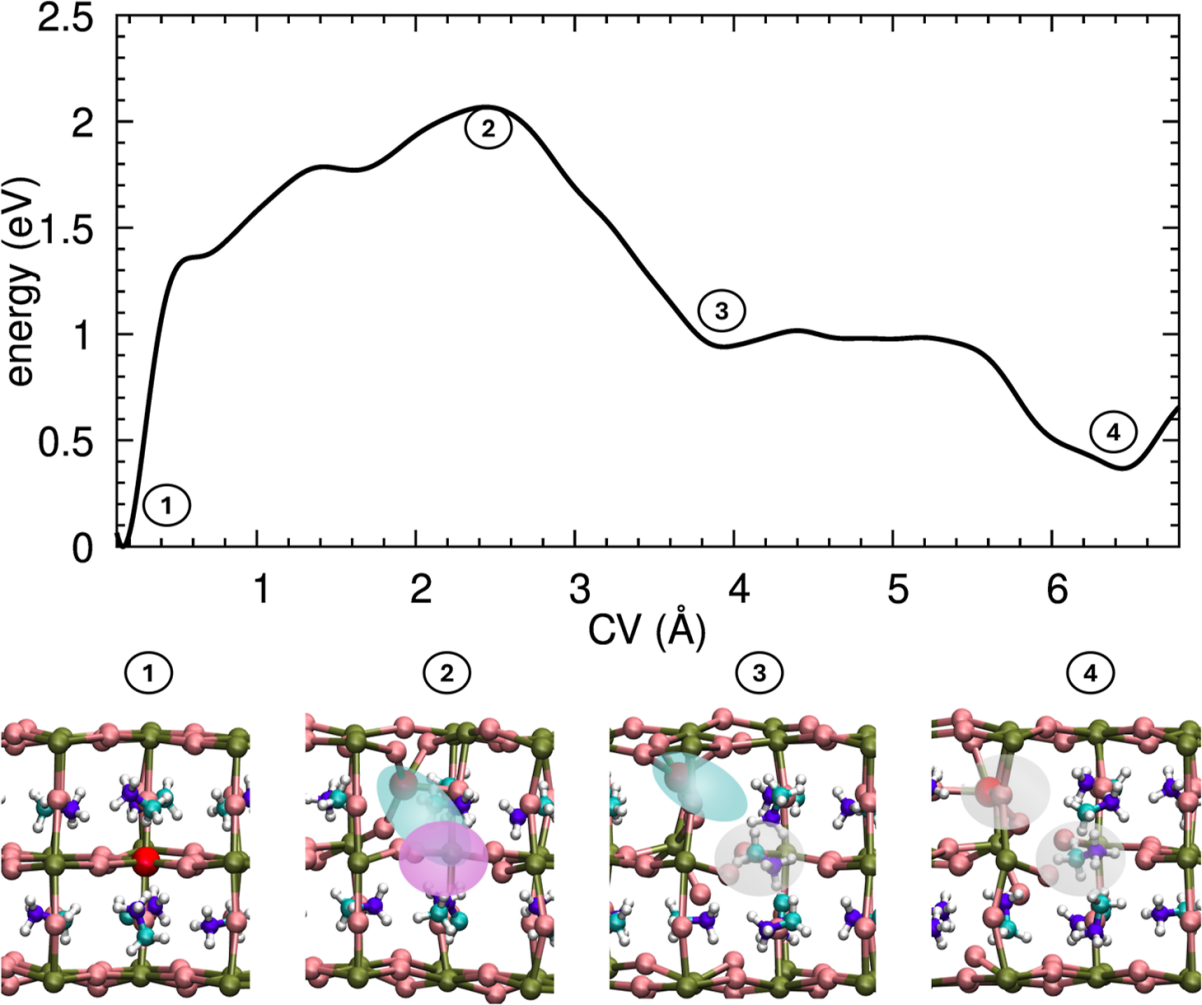
Formation of lead double antisite defects. Top panel: free energy surface (FES) for the displacement of a lead atom. Bottom panel: atomistic evolution: (1) pristine crystal; (2) transition state for Frenkel formation; (3) intermediate state involving *MA*_Pb_ antisite formation; (4) relaxation into the stable double antisite pair *Pb*_MA_ + *MA*_Pb_. Color code: biased-Pb (red), lattice Pb (green), I (brown), MA [H (white), C (light blue), N (dark blue)].

However, the postbarrier evolution does not lead to recombination. Unlike the monovalent cases where the bound state at *R*_*B*_ is a turning point for healing, this configuration for lead is chemically unstable. Around 4 Å, the system explores a shallow local minimum (Snapshot 3) corresponding to an intermediate state where the interstitial lead begins to interact strongly with the organic cage. This interaction drives the transformation of the Frenkel pair and the creation of a molecular antisite.

From this intermediate configuration, the system relaxes into a deep, stable basin located around 6.5 Å. Our simulations identify this final configuration (Snapshot 4) as a double antisite pair (*Pb*_MA_ + *MA*_Pb_), formed by the complete swapping of the lead atom into the A-site cage and the concurrent displacement of the methylammonium molecule into the inorganic vacancy. Energetically, this defect is metastable, lying ∼0.4 eV above the pristine crystal energy. Unlike the transient Frenkel pairs of I and MA, the lead double antisite is structurally trapped in this configuration by high reverse barriers, preventing the restoration of the perfect lattice. This finding highlights the lead sublattice as the most problematic component, as it provides a pathway for the formation of persistent, complex defects that are likely acting as persistent structural defects that may impact the material’s electronic quality.

### Long-Range Interaction

While the metadynamics simulations elucidate the kinetic mechanisms of defect formation and healing at the local scale, a rigorous thermodynamic assessment requires quantifying the interaction between the charged defects as a function of their separation distance. To address this, we extended our analysis to a significantly larger system composed of a supercell of 20 × 20 × 4 formula units and 9600 atoms. In this expanded configuration, we generated Frenkel defect pairs for the three constituent species, as well as the lead double antisite complex (*Pb*_MA_ + *MA*_Pb_). The defect centers were placed at increasing distances of up to 7 nm in a crystalline bulk equilibrated at room temperature (see bottom panel of Figure 4). The formation energies were subsequently evaluated via finite-temperature molecular dynamics as the difference between the defective and perfect crystal at the same temperature (see Supporting Information for details).

**Figure 4:**
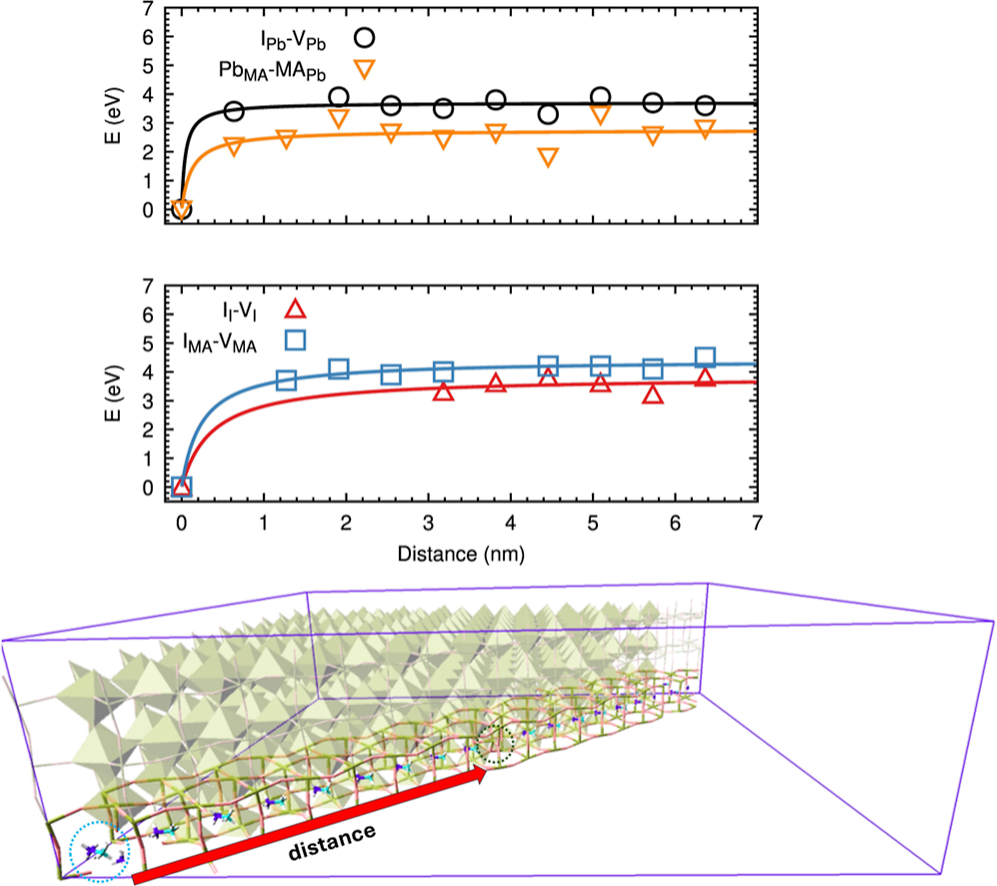
Top panel: formation energies of defect pairs calculated as a function of separation distance. Bottom panel: representation of the simulation box with the periodic MAPI crystal (9600 atoms) used to calculate the energy of Frenkel pairs; for clarity, only half of the crystal have been represented. The highlighted region shows a representative double antisite pair: *MA*_Pb_ (cyan circle) and *Pb*_MA_ (green circle) placed along a line of cubic PbI cells (corresponding to the ⟨110⟩ direction of the room temperature tetragonal crystal).

Figure 4 refers to room temperature and reports the computed energy profiles as a function of distance, *d*, at room temperature. Profiles for all different α defects exhibit a characteristic $E_{\alpha }\left(d\right)\approx E_{\alpha }^{\infty }\left(1-\frac{D_{\alpha }}{d+D_{\alpha }}\right)$ asymptotic behavior consistent with long-range Coulombic interaction between charged centers. In particular, for the iodine defects (red triangles in the middle panel), data points at short separations (<3 nm) are absent. This is a direct manifestation of the rapid self-healing mechanism: at these distances, the attractive Coulombic force overcomes thermal fluctuations, triggering the spontaneous annihilation of the *I*_i_ + *V*_I_ pair during equilibration. More detailed explanation on the dynamics of the iodine recombination can be found in Supporting Information, Section S2.3. A similar instability is observed for the methylammonium cation (blue squares), where stable defect pairs persist without recombining only beyond a critical separation threshold.

By combining the asymptotic formation energies (*E* ^*∞*^) extracted from these long-range profiles with the formation energies of the bound pairs (*E*_bound_), with the distance *R*_B_ identified in the previous section, we can define the thermodynamic dissociation energy as

1 $$\Delta E_{diss}=E^{\infty }-E_{bound}\left(R_{B}\right)$$

This value represents the minimum thermodynamic work required to fully separate the defect pair from its most stable bound configuration against the Coulombic attraction. Table 2 summarizes these energetic parameters for all considered species. For Iodine and Methylammonium, the dissociation costs are substantial (∼2.1 eV and ∼1.8 eV, respectively). This high energetic penalty confirms that under equilibrium conditions, the bound Frenkel pair is strongly favored over isolated defects, driving the self-healing process.

**Table 2:** Summary of defect energetic properties calculated in this work and comparison with literature ranges reported in Table 1

	literature	this work (MD)		
defect	barrier	barrier^a^	*E*_diss_	*R*_capt_
	(eV)	(eV)	(eV)	(nm)
*V*_I_ – *I*_i_	0.27–1.46	1.3 ± 0.1	2.1	3.3
*V*_MA_ – *MA*_i_	0.94–1.56	2.0 ± 0.2	1.8	1.4
*V*_Pb_ – *Pb*_i_	1.13^b^–2.15^b^	2.1 ± 0.2	0.7	0.4
*Pb*_MA_ – *MA*_Pb_	–	2.1	0.9	1.1

a The statistical uncertainties for the calculated barriers are evaluated via a reweighted block analysis (see Supporting Information).

b Includes values from non-MAPbI_3_ perovskite compositions.

The profiles of lead Frenkel defects reveal a distinct energetic hierarchy. We compared the standard Frenkel pair (*Pb*_*i*_ – *V*_*Pb*_, black circles) with the double antisite complex (*Pb*_*MA*_ + *MA*_*Pb*_, orange inverted triangles). The double antisite configuration consistently lies at a significantly lower energy (∼1.0 eV lower) compared to the Pb Frenkel pair throughout the separation range. Most importantly, among all the separated defect species analyzed, the double antisite pair exhibits the lowest plateau energy (∼2.7 eV).

Interestingly, Table 2 shows that lead complexes exhibit the lowest dissociation energies (∼0.7 eV for the Frenkel pair and ∼0.9 eV for the antisite). In principle, this weaker binding might suggest an easier separation compared to monovalent species. However, the actual probability of dissociation is strictly governed by the interplay between thermodynamics and kinetics. While I and MA have high dissociation costs, they possess high intrinsic mobility, allowing them to escape if the energy barrier is overcome (e.g., by electric fields). Conversely, the separation of Lead defects is severely limited by the low mobility of the lattice framework. Thus, despite the lower thermodynamic penalty for dissociation (*Pb*_*MA*_ – *MA*_*Pb*_), the antisite pair remains kinetically trapped in a bound or proximal state, acting as a localized recombination center rather than a source of mobile ions.

To further quantify the spatial extent of these interactions, we estimated the capture radius (*R*_*c*_) for each species, which spatially describes the extent of the interaction between the ions of the Frenkel pair. Conceptually, the formation of a Frenkel pair from an ideal crystal requires a total work equal to *E*_α_^∞^ to move an atom from its lattice site to an infinite distance from its resulting vacancy. We define *R*_*c*_ as the separation distance at which the formation energy reaches 90% of this asymptotic plateau value (see Methods).

Accordingly, the interaction potential between the vacancy and interstitial is characterized by two parameters: the interaction energy (*E*_α_^∞^) and its spatial extent (*R*_*c*_). These parameters directly influence the recombination dynamics; for a constant defect mobility, the recombination process becomes more efficient as *R*_*c*_ and *E*_α_^∞^ increase. Beyond *R*_*c*_, the defects behave as noninteracting entities, while below *R*_*c*_, the interaction is increasingly dominated by Coulombic attraction.

Comparing the species, we find that monovalent Frenkel pairs exhibit a substantial capture range, estimated at around 3.3 nm for Iodine and 1.4 nm for MA. The large Iodine capture radius was further validated by finite-temperature Molecular Dynamics simulations at 300 K. When starting from *V*_*I*_-*I*_*i*_ pair with defect separations *d* < *R*_*c*_, we observed rapid recombination within approximately 100 ps. For initial separations *d* > *R*_*c*_, the *V*_*I*_ and *I*_*i*_ defects diffuse independently, recombining only when stochastic diffusion brings them within the critical distance *d* < *R*_*c*_. The probability of such events increases over longer time scales or at higher temperatures, which enhance defect mobility.

Conversely, the standard lead Frenkel pair (*V*_*Pb*_^2‑^ – *Pb*_*i*_^2+^) shows a remarkably short interaction range (∼0.4 nm). This spatial confinement is attributed to the divalent nature of the defect: the high charge density induces strong local lattice polarization, leading to efficient screening that steepens the potential decay. Nevertheless, when the system relaxes into the double antisite complex (*Pb*_*MA*_^+^ – *MA*_*Pb*_^‑^), the interaction range expands to ∼1.1 nm. This increase reflects the reduction of the effective charge to a monovalent state (±1), which results in an interaction range comparable to that of other monovalent Frenkel pairs.

To further clarify the physical interpretation of the interaction model and the role of dielectric screening, the effective charge reproducing the long-range interaction energy can be estimated analytically (see Supporting Information S2.3 for the full derivation). The effective charge is consistently lower than the nominal charge (*Q*_eff_ < *Q*_nominal_), as expected from dielectric screening and lattice polarization. These *Q*_eff_ values strictly follow the trend of the interaction energy *E*_α_^∞^. The strongest screening (*Q*_eff_/*Q* ≈ 0.28) occurs for the highly charged divalent lead cation, while monovalent ions show a screening factor of ≈0.7. Notably, the double antisite complex exhibits the lowest absolute effective charge, fully consistent with its lower interaction energy *E*_α_^∞^ and its stable nature from an electrostatic perspective.

This comprehensive analysis provides robust thermodynamic confirmation that the irreversible evolution of lead defects observed in metadynamics is not only kinetically accessible but also leads to the most energetically favorable long-lived defect state in the crystal.

## Conclusions

In this work, we combined classical molecular dynamics and metadynamics to investigate the energetics and atomistic mechanisms governing the formation and evolution of Frenkel defects in stoichiometric methylammonium lead iodide at room temperature. A key strength of our approach lies in the synergetic use of simulations across different length scales. While metadynamics allowed us to sample local kinetic barriers in standard supercells (3072 atoms), the extension to large-scale models (9600 atoms) enabled the rigorous mapping of defect interactions up to a separation distance of 7 nm. To the best of our knowledge, such an extended interaction range has not been previously explored in atomistic simulations of hybrid perovskites, allowing us to quantify thermodynamic dissociation energies and capture radii (Table 2).

Our analysis reveals that the monovalent species (I and MA) exhibit intrinsic resilience. Iodine Frenkel defects display the lowest formation barrier (1.3 eV) and the largest capture radius (interaction range). Similarly, the methylammonium cation requires a higher formation barrier (∼2.0 eV) but is characterized by a shorter interaction range. Despite these differences, simulations show that for both species, local perturbations trigger a rapid self-healing response, realized either through direct recombination or a concerted self-diffusion mechanism. Crucially, the complete dissociation of the Frenkel pair into isolated defects is energetically disfavored, requiring an additional energetic cost of ∼2 eV. This high dissociation barrier effectively confines the defects, promoting their annihilation over their separation. Consequently, our results suggest that the high concentration of mobile halide defects often observed in real samples^55^ is likely not an intrinsic property of the equilibrated crystal bulk. Instead, it arguably originates from microstructural imperfections (surfaces, grain boundaries^56,57^ ), nonequilibrium growth conditions, or external biases.

In contrast, the divalent lead sublattice exhibits different behavior. Although the formation of a Pb Frenkel pair involves a comparable energy barrier (2.1 eV), this defect does not undergo rapid recombination. Instead, the local instability triggers a spontaneous cation exchange, evolving into a metastable double antisite complex (*Pb*_*MA*_ + *MA*_*Pb*_). This configuration is robust against recombination and forms a structurally stable complex. While our classical molecular dynamics simulations capture the structural relaxation, this persistent defect configuration is expected to act as a deep electronic trap state. Recent first-principles investigations on structurally analogous defects in FAPbI_3_ ^36^ demonstrated that bound double antisite Pb_*FA*_ – FA_*Pb*_ complexes introduce deep levels within the bandgap. Since the relevant electronic band edges in these perovskites are predominantly determined by the inorganic Pb–I framework, the deep-trap nature of these Pb-related complexes is expected to remain consistent irrespective of the specific organic cation substitution. The significance of Pb_*MA*_ antisite has been previously reported as a defect that can spontaneously form at crystalline surfaces during crystallization.^58^ Here, we identify a novel mechanism for the formation and stabilization of double antisites within the pristine crystal bulk.

Collectively, these findings provide a mechanistic rationale for the defect tolerance of hybrid perovskites, driven by the rapid healing of the halide and organic sublattices, while identifying the irreversible relaxation of the inorganic lead framework as a critical source of degradation and trap states.

## Methods

### Computational Details and Potential

All molecular dynamics (MD) simulations were carried out using the LAMMPS^59^ code interfaced with the PLUMED 2.8 library.^60^ Interatomic interactions were modeled using the fixed-charge MYP0 potential developed by Mattoni et al.,^44^ which has been shown to accurately reproduce key properties of hybrid perovskites, including lattice dynamics, phase transitions, vibrational spectra,^45^ and defect dynamics.^46,48,61^ Several studies have successfully employed the MYP0 force field to investigate defect-related properties, including screening,^62^ mobility, migration barriers and point-defect morphology^46^ and the interaction of defects with surfaces,^61^ grain boundaries,^48^ ferroelastic domains.^47^ In all these cases, the model yielded results in consistent qualitative agreement with both DFT data and experimental findings. A benchmarking of the force field’s reliability against available first-principles DFT data, including a quantitative comparison of the absolute defect formation energies (Table S1), is provided in the Supporting Information.

Further details on the parametrization of the MYP0 are reported elsewhere.^44^ To investigate the spontaneous formation of Frenkel defects, we employed classical metadynamics (MetaD)^42,63^ on a supercell containing 3072 atoms (256 formula units).

Simulations were conducted in the isothermal–isobaric (*NPT*) ensemble at 300 K using a time step of 1 fs. Long-range electrostatics were treated via the PPPM method. Complete details on the simulation protocol, equilibration procedures, force-field, and metadynamics parameters are provided in the Supporting Information.

### Recombination and Long-Range Interaction

The long-range interaction within an isolated Frenkel pair was studied using larger supercells containing 9600 atoms to minimize finite-size effects. Frenkel pairs were generated at systematically increasing separation distances *d* (details on generation and equilibration in SI).

The formation energy *E*_*F*_ (*d*) was evaluated via finite-temperature MD as the difference between the average potential energies of the defective and bulk crystals (defined as eq S2 in the Supporting Information).

The distance-dependent formation energies were then fitted using an effective electrostatic-like interaction model of the form

2 $$f_{\alpha }\left(d\right)=E_{\alpha }^{\infty }\left(1-\frac{D_{\alpha }}{d+D_{\alpha }}\right)$$

where *d* is the defect separation, the subscript α denotes the defect type, and *E*_α_^∞^ and *D*_α_ are adjustable parameters fitted to the atomistic data. From this, we quantified a capture radius *R*_*c*_, defined as the distance at which the interaction energy reaches a fraction (1 – *f*) = 90% of its plateau (eq 4 Supporting Information). Details for the recombination dynamics of iodine can be found in Supporting Information.

## Supplementary Material





## Notes

Supporting Information (PDF): Extended computational details, metadynamics parameters, derivation of the capture radius, and detailed analysis protocols (PDF)
Simulation Data (TGZ): An archive containing LAMMPS input scripts, PLUMED configuration files for metadynamics, and input files for recombination and long-range interaction simulations (ZIP)

## Abbreviations

Methylammonium Lead Iodide: (MAPI or MAPbI_3_)
Molecular Dynamics: (MD)
Free Energy Surface: (FES)
Methylammonium: (MA)
Collective Variable: (CV).
