# Supplementary material for: Finite-Temperature Evolution of Frenkel Defects in Hybrid Perovskites: Healing and Lead-Methylammonium Antisite Pairs
Source: ACS Appl Mater Interfaces. 2026 Apr 9;18(15):22071–80. doi: 10.1021/acsami.6c02743 (PMC13107386; doi:10.1021/acsami.6c02743)
Supplement: Supplementary file 1 [file am6c02743_si_001.pdf]

Supporting Information

Finite-Temperature Evolution of Frenkel Defects in Hybrid Perovskites: Healing and Lead-Methylammonium Antisite Pairs

Jgor Pensè Schone[1,2], Simone Argiolas[1,2], Alessio Gagliardi[3], Gohar Ali Siddiqui[3], Alessio Filippetti[1,2], and Alessandro Mattoni[*1]

[1]CNR – Istituto Officina dei Materiali (IOM), Cagliari, Cittadella Universitaria, Monserrato, (CA) 09042, Italy

[2]Dipartimento di Fisica, Università degli Studi di Cagliari, Cittadella Universitaria, Monserrato, (CA) 09042, Italy

[3]Chair of Simulation of Nanosystems for Energy Conversion, Department of Electrical Engineering, TUM School of Computation, Information and Technology, Atomistic Modeling Center (AMC), Munich Data Science Institute (MDSI), Technical University of Munich, Garching, Germany

*Email: mattoni@iom.cnr.it

S1 Benchmarking of MYP0 force field

The MYP0 potential is a classical fixed-charge model developed to accurately reproduce the structural, vibrational, and dielectric properties of $MAPbI_3$, where the ionic component of polarization is dominant. While a non-polarizable classical force field cannot explicitly account for dynamic changes in atomic oxidation states or electronic rearrangements during bond breaking, we have extensively benchmarked its predictions against available first-principles density functional theory (DFT) data to ensure its reliability in describing defect chemistry.

S1.1 Defect Energies

To ensure the reliability of the MYP0 fixed-charge model in describing defect chemistry, we extensively benchmarked its predictions against available first-principles density functional theory (DFT) data. Specifically, we compared the formation energy (E_F) of intimate Frenkel pairs extracted from the long-range interaction profiles (Figure 4 of the main text), calculated at 300 K using the MYP0 potential at the distance of the bound radius (R_B), against the most accurate first-principles results available in the literature (e.g., HSE06+SOC). The results are summarized in Table S1.

Table S1: Comparison of Frenkel pair formation energies calculated via finite-temperature MD (MYP0, 300 K at the bound radius R_B) and static first-principles DFT (0 K) from literature.

**Defect species**	**MYP0 E_F (300 K, R_B)**	**DFT (0 K)**
Iodine (I)	1.5 eV	1.36 eV[1]
Methylammonium (MA)	2.0 eV	1.56 eV[2]
Lead (Pb)	3.0 eV	2.15 eV[3]

As shown in Table S1, while the fixed-charge MYP0 potential tends to overestimate absolute formation energies by approximately 20–50%, it accurately reproduces the stability hierarchy (I < MA < Pb) observed in first-principles calculations. This systematic overestimation is a known characteristic of non-polarizable classical models, which lack explicit electronic relaxation and dynamic polarizability. Furthermore, the MD values at

300 K naturally account for thermal lattice distortions and vibrational entropy, which are crucial for a realistic thermodynamic description of the soft perovskite lattice.

S1.2 Defect Morphology and Migration

In addition to energetics, the MYP0 model accurately captures the complex structural morphologies of point defects. For instance, our molecular dynamics simulations show that the potential spontaneously predicts the characteristic dumbbell configuration for the iodine interstitial (I_i^-). This geometry, involving a covalent-like $I - I$ bond within the lattice, is in excellent structural agreement with established DFT literature [4, 5], confirming that the force field correctly describes the local lattice distortions associated with defect formation.

Secondly, the potential demonstrates remarkable quantitative agreement in predicting kinetic properties and migration pathways. As shown in Figure S1, the maximum energy predicted by our model closely matches the ab initio values reported by Meloni et al.[6] for equatorial-equatorial migration. Furthermore, the comparison with the full diffusion path reported by Eames et al.[7] confirms that the model potential's reliability falls within the typical accuracy range of different ab initio methods. Beyond static analysis, the activation energy obtained from dynamics (Delugas et al.[8]) is also in close proximity to the experimental value (0.1 eV).

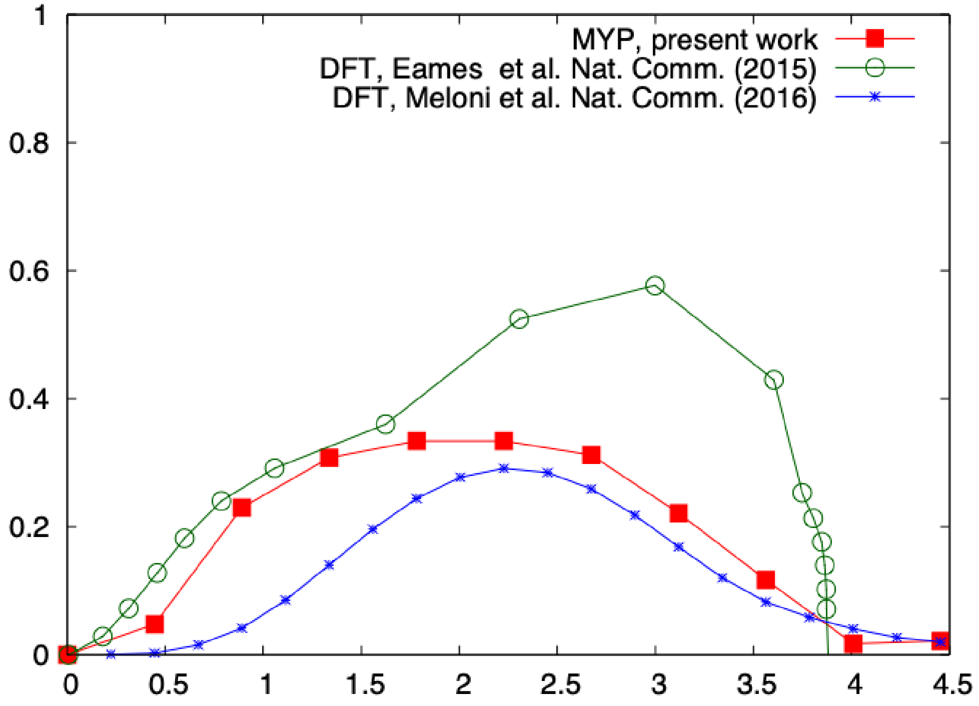

Figure S1: Static energy (eV) profile for vacancy diffusion (equatorial-equatorial) along the reaction path (Å), calculated by the MYP0 potential (red squares) and compared to ab initio data from Meloni et al. (blue asterisks) and Eames et al. (green circles).

Collectively, these benchmarks confirm that while MYP0 may exhibit intrinsic rigidities in evaluating absolute defect formation energies, it provides a highly reliable and robust description of the relative energetic landscape, defect geometries, and mechanistic pathways. Such fidelity is essential for investigating the formation, migration, and self-healing of Frenkel defects in hybrid perovskites with atomistic precision.

S2 Computational Methods

Here we provide extended details regarding the molecular dynamics simulation protocol adopted in the main text. Simulations were performed on a supercell containing 256 formula units, corresponding to 3072 atoms. Periodic boundary conditions were applied in all directions. The equations of motion were integrated using a timestep of 1 fs. Long-range electrostatic interactions were treated using the particle-particle particle-mesh (PPPM) method with a real-space cutoff of 10 Å, while short-range interactions

were described using a hybrid Lennard–Jones, Buckingham, and Coulombic potential, consistent with the functional forms and parameterization of the MYP0 force field[9]. The system was gradually annealed to a target temperature of 300 K over a timescale of several hundred picoseconds, followed by an equilibration phase in the isothermal–isobaric (NPT) ensemble. Subsequently, metadynamics simulations were performed, resulting in total simulation times on the order of one nanosecond.

S2.1 Metadynamics and Defect Energetics

The metadynamics simulations reported in this work represent the final stage of an extensive methodological benchmarking. To ensure the reliability of the identified mechanisms and energy barriers, we performed approximately 50 independent simulations, totaling more than 100 ns of cumulative sampling.

During this phase, we systematically explored the parameter space by varying the hill height (from 0.5 to 2.5 kcal/mol), the deposition pace from 100 to 500 steps, and the Gaussian width (σ from 0.1 to 0.2 Å). The choice of σ was physically grounded in the average thermal fluctuations of the atoms within the $MAPbI_3$ lattice at 300 K [10]. We observed that the migration pathways were consistent across this entire range, confirming that the mechanisms are intrinsic physical features of the potential energy surface rather than artifacts of the biasing parameters.

We specifically optimized the deposition rate to ensure an adiabatic regime, allowing the soft perovskite lattice to undergo the necessary cooperative relaxations and avoiding hysteresis. To further ensure statistical robustness, we performed a reweighted block analysis on the converged trajectories, identifying clear error plateaus (see Section S2.2) that confirm effective sampling beyond the system's correlation time.

The Frenkel pair formation process was driven by a Collective Variable (CV) defined as the distance between the biased atom (I, Pb, MA) and its initial equilibrium position. For methylammonium (MA), the collective variable was defined analogously as a distance, but utilizing the mass-weighted center of mass of the biased molecule as the reference point. The initial reference position of the molecule was defined as the time-averaged center of

mass over 50 configurations prior to the initialization of the metadynamics simulation. This choice reduces the influence of thermal fluctuations and molecular reorientations on the definition of the CV. Our selection of the distance-based CV is grounded in its effectiveness for these systems. Despite being a simple descriptor, this CV is highly effective at allowing the system to cooperatively relax into minimum energy configurations without imposing non-physical geometric constraints. The reliability of this choice is validated by its ability to spontaneously reproduce the characteristic iodine dumbbell configuration, widely recognized in the literature as the minimum energy geometry for this defect. Crucially, this choice does not artificially constrain the surrounding environment or the internal degrees of freedom of the molecules, enabling the observation of complex secondary effects, such as the spontaneous reorientations during the exchange mechanisms reported in the main text.

The Gaussian hill parameters for iodine and lead were set to a height of 2.0 kcal/mol, a width (σ) of 0.2 Å, and a deposition pace of 500 steps (0.5 ps). Optimized metadynamics parameters were adopted for the MA molecule, with a Gaussian width of $\sigma = 0.2$ Å, a height of 1.0 kcal/mol, and a deposition pace of 200 MD steps. These adjustments were necessary to achieve consistent sampling and free energy accuracy across different defect types. The formation energy barrier was calculated from the reconstructed Free Energy Surface (FES) as the difference between the global minimum (pristine state) and the transition state maximum.

We considered the dissociation pathway only for methylammonium (MA). In this case, the vacancy and the corresponding interstitial were driven to large separations, effectively preventing immediate recombination and enabling the analysis of the dissociated defect state.

For iodine Frenkel defects, due to the high intrinsic mobility of iodine ions, the dissociation and recombination dynamics were investigated instead using an alternative approach, which is described in detail in the following section.

To achieve this separation, a lower-wall restraint was applied to the distance collective variable. The wall acts as an energetic barrier on the molecular species closest to the

vacancy, preventing its collapse back into the vacant site, while not directly biasing the displaced interstitial species, which remains free to diffuse during the dynamics.

The lower-wall bias potential was defined following the standard formulation implemented in PLUMED [11]:

$$V_{\text{wall}}(s) = \begin{cases} \kappa \left(\dfrac{s - s_0 + o}{\varepsilon} \right)^e, & s < s_0 - o \\ 0, & s \geq s_0 - o \end{cases} \tag{S1}$$

where s is the distance collective variable, s_0 is the cutoff position of the wall, κ is the force constant of the wall, and e controls the steepness of the restraint. In the present work, the wall parameters were set to $s_0 = 4.25$ Å, $\kappa = 500.0$, and $e = 2$. The scaling factor and offset were set to $\varepsilon = 1$ and $o = 0$, respectively, such that the wall reduces to a simple power-law restraint.

S2.2 Metadynamics Convergence and Block Analysis

To ensure the convergence of the metadynamics simulations and establish the robustness of the calculated free energy barriers, we monitored the temporal evolution of the Free Energy Surface (FES) and performed a reweighted block analysis. The stationarity of the FES was verified by comparing energy profiles at different simulation stages, confirming that the depth of the metastable basins and the height of the transition states reached a stable regime once the bias potential effectively compensated the underlying energy landscape.

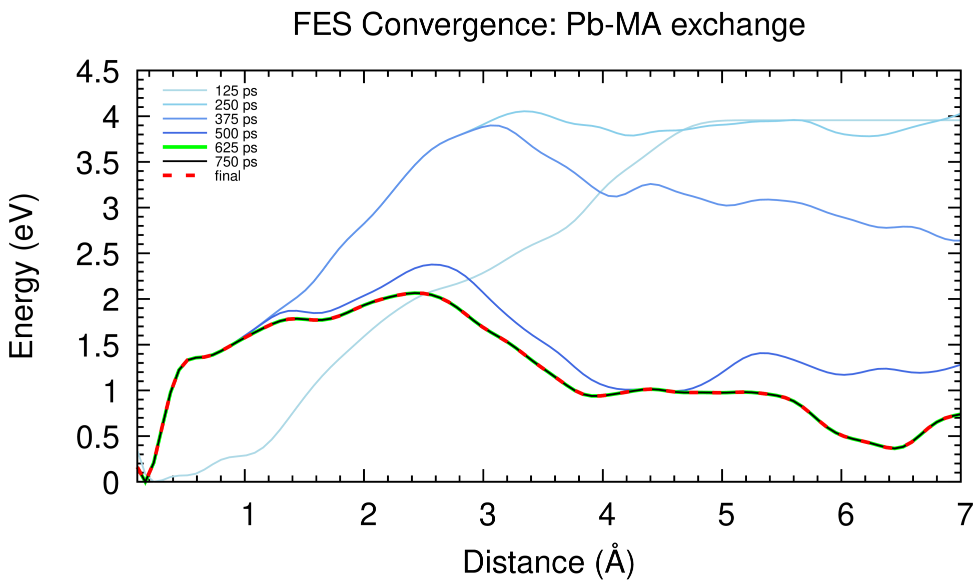

Figure S2: Estimates of the free energy as a function of the distance calculated every 250 Gaussians deposited.

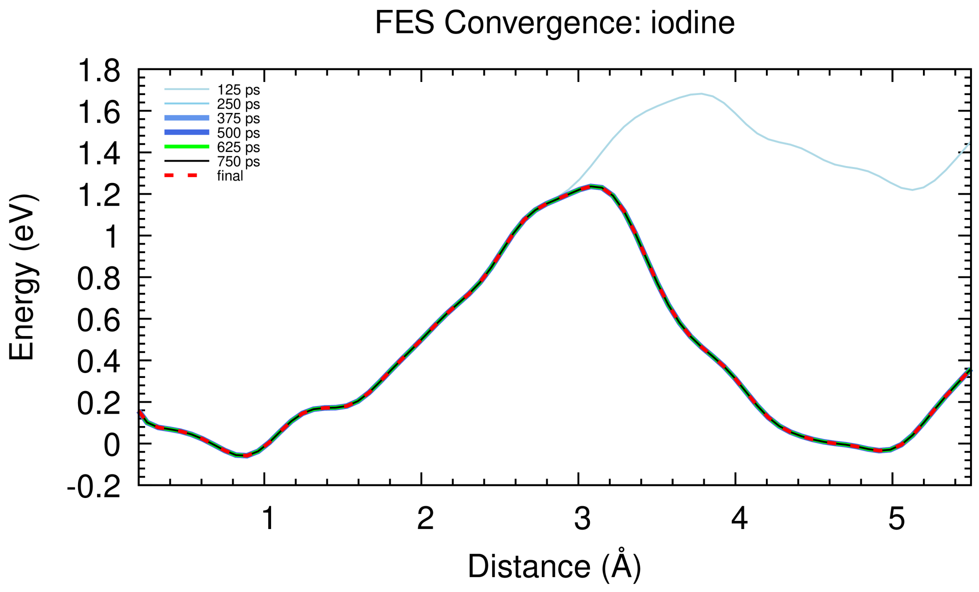

Figure S3: Estimates of the free energy as a function of the distance calculated every 250 Gaussians deposited.

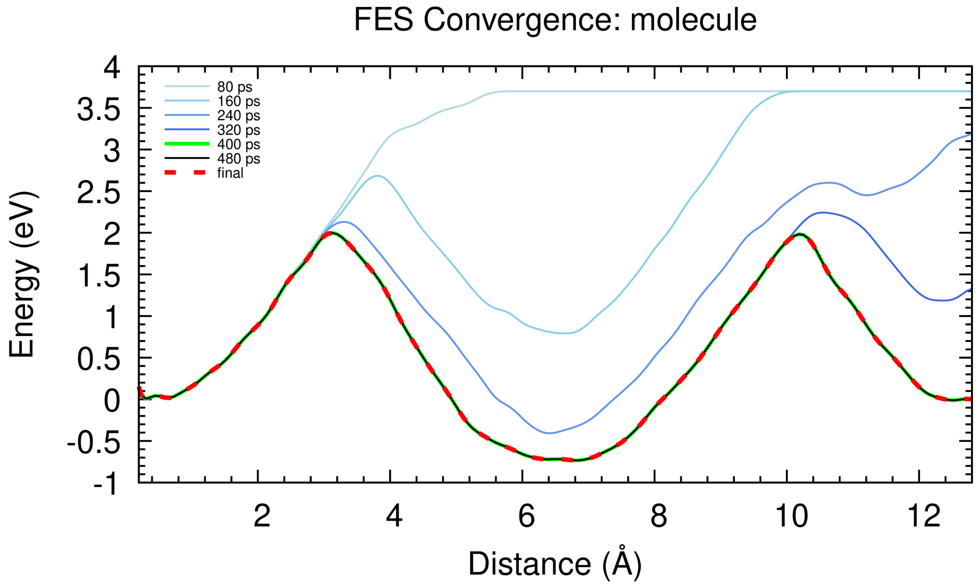

Figure S4: Estimates of the free energy as a function of the distance calculated every 400 Gaussians deposited.

To assess quantitative convergence and determine the statistical uncertainty of the calculated free-energy barriers, we performed a reweighted block analysis on the converged trajectories. By monitoring the statistical error as a function of the block size (see Figure S5), we identified a clear plateau for all investigated species. This behavior indicates that the sampling effectively overcomes the intrinsic correlation time of the system, ensuring that the reconstructed FES is statistically independent and reliable.

The raw statistical errors derived from the plateau values range between 0.08 and 0.13 eV. Based on these results, we adopted a conservative uncertainty of ± 0.1 eV for the iodine species and ± 0.2 eV for both lead and methylammonium in Table 2 of the main text. This choice accounts for both the sampling variance and the inherent systematic accuracy of the fixed-charge potential model, providing a robust confidence interval of approximately 10% for the reported energy barriers.

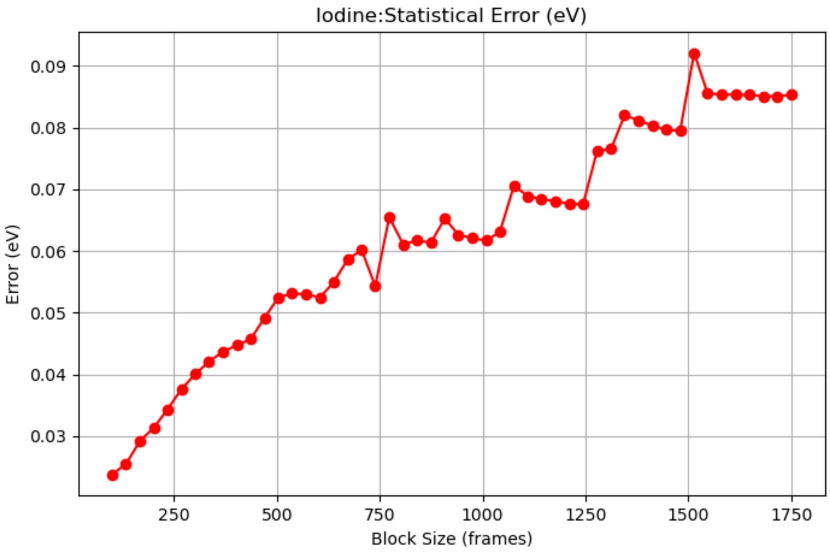

Figure S5: Quantitative error estimation via reweighted block analysis for Iodine. The plateau indicates that the block size is sufficient to overcome the correlation time of the sampling.

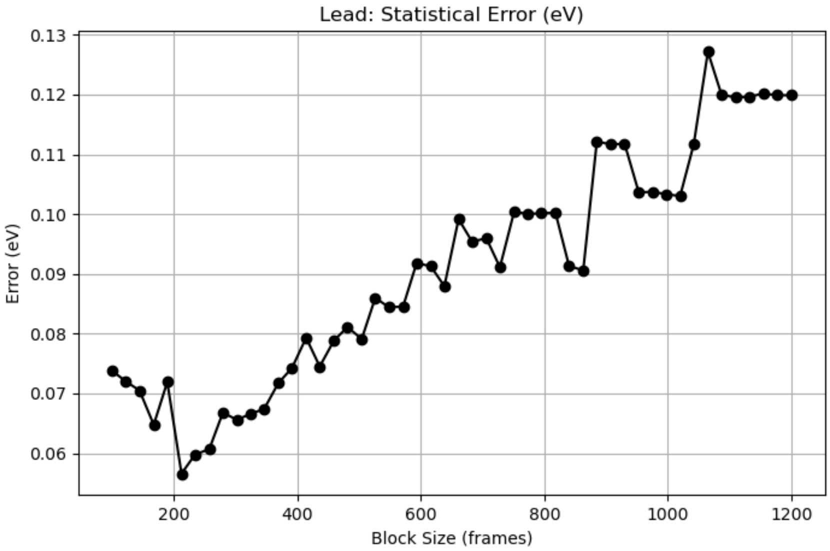

Figure S6: Quantitative error estimation via reweighted block analysis for Lead. The plateau indicates that the block size is sufficient to overcome the correlation time of the sampling.

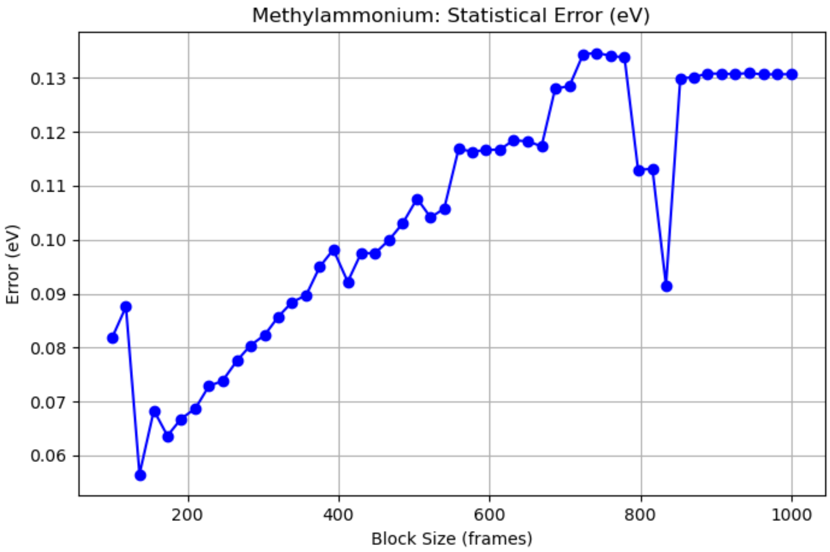

Figure S7: Quantitative error estimation via reweighted block analysis for Methylammonimum. The plateau indicates that the block size is sufficient to overcome the correlation time of the sampling.

S2.3 Recombination and long-range interaction

We chose to analyze the long range interaction in 20x20x4 formula units supercells, containing 9600 atoms. This is due to minimize finite-size effects and to properly capture long-range interactions under periodic boundary conditions. We considered three distinct Frenkel pair configurations, as well as a double antisite defect involving Pb-MA and MA-Pb exchanges.

Specifically, Frenkel pairs were created by displacing one atom of the defect species along the $\langle 110 \rangle$ crystallographic direction of the tetragonal crystal. The defect separation varied from a minimum distance of 0.6 nm up to approximately 7.0 nm, accounting for the minimum-image convention. This protocol enabled a systematic exploration of defect interactions over a wide range of distances within a single supercell. Although the Frenkel pair energy may depend on the crystallographic direction, to reduce computational cost, we focused exclusively on the $\langle 110 \rangle$ direction, which corresponds to the orientation perpendicular to the faces of the Pb-I cubic cages.

For each defect configuration, the system was first structurally stabilized at low tem-

perature. The system was then gradually annealed to 300 K using constant-pressure molecular dynamics in the anisotropic NPT ensemble, with a timestep of 1 fs. Temperature and pressure were controlled using a Nosé–Hoover thermostat and barostat, allowing independent fluctuations of the cell vectors. Subsequently, production simulations of 0.1 ns were performed at the target temperature.

The formation energy (E_F) of each Frenkel pair depends on the separation d between the vacancy and the interstitial and is evaluated by averaging the total energy over the last 50000 MD steps of the equilibration phase for both the perfect bulk (E_B) and defective systems (E_D), according to

$$E_F(d) = \langle E_D(d) \rangle - \langle E_B \rangle. \tag{S2}$$

The distance-dependent formation energies were fitted using an effective electrostatic-like interaction model of the form:

$$f_\alpha(d) = E_\alpha^\infty \left(1 - \frac{D_\alpha}{d + D_\alpha}\right), \tag{S3}$$

where d is the defect separation, the subscript α denotes the defect type, and E_α^∞ and D_α are adjustable parameters fitted to the atomistic data. Here, E_α^∞ represents the asymptotic formation energy corresponding to fully separated vacancy and interstitial defects, while D_α controls the interaction range.

We quantified a capture radius R_c, defined as the distance at which the formation energy reaches a fraction $(1-f)$ of the asymptotic value E_α^∞. Solving the model equation yields:

$$R_c = \frac{1-f}{f} D_\alpha \tag{S4}$$

In the present work, we adopt $(1 - f) = 90\%$. The chosen functional form captures the screened electrostatic nature of charged defect interactions.

To quantify the combined effects of dielectric screening and lattice relaxation on the defect interactions, we evaluated the effective charge (Q_{eff}) of the defects starting from

the nominal ionic partial charges of the MYP0 force field. The nominal charge of an interstitial defect equals the partial ionic charge of the corresponding species as defined by the MYP0 force field (I: $-1.13\ e$, Pb: $+2.03\ e$, MA: $+1.36\ e$), while the corresponding vacancy takes the exact opposite charge[9].

By leveraging the long-range energetics data, it is possible to estimate the effective charge Q_{eff} that reproduces the long-range interaction energy for $d > 0$. The interaction energy can be expressed as:

$$E(d) = E_{\alpha}^{\infty} - \frac{Q_{eff}^2}{4\pi\varepsilon_0 d} = E_{\alpha}^{\infty}\left(1 - \frac{D_\alpha}{D_\alpha + d}\right) \tag{S5}$$

where D_α and E_{α}^{∞} are the model fit parameters, and ε_0 is the vacuum permittivity. Q_{eff} can be derived as a function of the distance d:

$$Q_{eff}^2(d) = 4\pi\varepsilon_0 E_{\alpha}^{\infty}\frac{D_\alpha d}{D_\alpha + d} \tag{S6}$$

In our analysis, Q_{eff} is evaluated at $d = R_B$ (the radius of the Frenkel pair in the bound state). The effective charges obtained for the different defect species are summarized in Table S2.

Table S2: Nominal charges ($|Q_{nominal}|$), fit parameters (E_{α}^{∞} and D_α), and derived effective charges (Q_{eff}) for the investigated defect pairs. The ratio Q_{eff}/Q highlights the magnitude of the dielectric screening.

**Species**	$\lvert Q_{nominal}\rvert$ **(e)**	E_{α}^{∞} **(eV)**	D_α **(nm)**	Q_{eff} **(e)**	Q_{eff}/Q
Iodine (I)	1.13	3.83	0.367	0.80	0.71
Iodine (I)	1.13	3.83	0.367	0.80	0.71
Lead (Pb)	2.03	3.79	0.045	0.58	0.28
Methylammonium (MA)	1.36	4.42	0.242	0.88	0.67
Pb_{MA}-MA_{Pb}	0.67	2.75	0.123	0.50	0.77

As expected due to dielectric screening, the effective value is always lower than the

nominal one ($Q_{eff} < Q_{nominal}$). The Q_{eff} values follow the same order as the interaction energy E_{α}^{∞}. The strongest screening ($Q_{eff}/Q \approx 0.28$) is observed for the divalent lead cation, while for monovalent ions, the screening factor is ≈ 0.7.

The minimum effective charge is found for the double antisite complex. This result is consistent with the fact that this complex has the lowest E_{α}^{∞}. This analysis further clarifies the physical interpretation of the fitting model by linking it to screening and long-range interaction energy, supporting the stability of the double antisite from an electrostatic perspective.

The recombination dynamics of iodine Frenkel pairs were further investigated using simulation cells comparable in size to those employed in the metadynamics simulations. The system was initialized with a vacancy-interstitial separation of approximately 3.0 nm, corresponding to a regime in which the two defects interact only weakly. The system was then equilibrated and evolved at the target temperature of 300 K.

At such separations, the process is kinetically limited by the lattice potential landscape: the vacancy and interstitial are separated by energy barriers and must undergo thermally activated diffusion to approach each other. Consequently, the defects do not simply collapse; they require sufficient thermal energy and time to migrate across the lattice until they enter the capture radius. The simulations therefore capture the intrinsic recombination dynamics at finite temperature, allowing the defects to either diffuse back into a bound configuration and recombine or remain spatially separated over the simulation timescale.

Molecular dynamics simulations were performed for a total duration of 0.6 ns with a timestep of 1 fs. During the simulation, defect tracking was carried out through an *in-house* post-processing script based on the local coordination environment of the lead sublattice. Specifically, the position of the iodine vacancy (V_I) was identified as the center of mass (COM) of the undercoordinated Pb atoms (coordination number < 6). Conversely, the iodine interstitial (I_i) was localized via the COM of the overcoordinated Pb atoms (coordination number > 6). A cutoff radius of 5.5 Å was employed to analyze the dynamical interaction range between the defect centers.

From this analysis, configurations corresponding to the onset of recombination events were extracted. These configurations were then used as starting points for additional MD simulations, in which atomic configurations and energies were saved every 10 fs, instead of the 100 fs interval employed in the original trajectories. By restricting the dynamics to the spatial region directly involved in the recombination process and re-propagating the trajectories from these configurations, we obtained a clearer and more resolved description of the recombination mechanism. This strategy also allowed for more accurate statistical averaging of the energetic quantities associated with the recombination process. As discussed in the main text, this high-resolution analysis confirmed that once the defects stochastically diffuse within the critical capture radius ($d < R_c$), the actual recombination event occurs rapidly, typically within an approximately 100 ps timeframe.

Bibliography

(1) Meggiolaro, D.; Mosconi, E.; De Angelis, F. Formation of Surface Defects Dominates Ion Migration in Lead-Halide Perovskites. *ACS Energy Lett.* **2019**, *4*, 779–785, DOI: 10.1021/acsenergylett.9b00247.

(2) Chen, B.; Li, T.; Dong, Q.; Mosconi, E.; Song, J.; Chen, Z.; Deng, Y.; Liu, Y.; Ducharme, S.; Gruverman, A.; Angelis, F. D.; Huang, J. Large Electrostrictive Response in Lead Halide Perovskites. *Nature Mater* **2018**, *17*, 1020–1026, DOI: 10.1038/s41563-018-0170-x.

(3) Jung, Y.-K.; Abdulla, M.; Friend, R. H.; Stranks, S. D.; Walsh, A. Pressure-Induced Non-Radiative Losses in Halide Perovskite Light-Emitting Diodes. *J. Mater. Chem. C* **2022**, *10*, 12560–12568, DOI: 10.1039/D2TC01490D.

(4) Mosconi, E.; Meggiolaro, D.; Snaith, H. J.; Stranks, S. D.; Angelis, F. D. Light-Induced Annihilation of Frenkel Defects in Organo-Lead Halide Perovskites. *Energy Environ. Sci.* **2016**, *9*, 3180–3187, DOI: 10.1039/C6EE01504B.

(5) Meggiolaro, D.; Motti, S. G.; Mosconi, E.; Barker, A. J.; Ball, J.; Andrea Riccardo Perini, C.; Deschler, F.; Petrozza, A.; De Angelis, F. Iodine Chemistry Determines the Defect Tolerance of Lead-Halide Perovskites. *Energy Environ. Sci.* **2018**, *11*, 702–713, DOI: 10.1039/C8EE00124C.

(6) Meloni, S.; Moehl, T.; Tress, W.; Franckevičius, M.; Saliba, M.; Lee, Y. H.; Gao, P.; Nazeeruddin, M. K.; Zakeeruddin, S. M.; Rothlisberger, U.; Graetzel, M. Ionic Polarization-Induced Current–Voltage Hysteresis in CH3NH3PbX3 Perovskite Solar Cells. *Nat Commun* **2016**, *7*, 10334, DOI: 10.1038/ncomms10334.

(7) Eames, C.; Frost, J. M.; Barnes, P. R. F.; O'Regan, B. C.; Walsh, A.; Islam, M. S. Ionic Transport in Hybrid Lead Iodide Perovskite Solar Cells. *Nat Commun* **2015**, *6*, 7497, DOI: 10.1038/ncomms8497.

(8) Delugas, P.; Caddeo, C.; Filippetti, A.; Mattoni, A. Thermally Activated Point Defect Diffusion in Methylammonium Lead Trihalide: Anisotropic and Ultrahigh Mobility of Iodine. *J. Phys. Chem. Lett.* **2016**, *7*, 2356–2361, DOI: 10.1021/acs.jpclett.6b00963.

(9) Mattoni, A.; Filippetti, A.; Saba, M. I.; Delugas, P. Methylammonium Rotational Dynamics in Lead Halide Perovskite by Classical Molecular Dynamics: The Role of Temperature. *J. Phys. Chem. C* **2015**, *119*, 17421–17428, DOI: 10.1021/acs.jpcc.5b04283.

(10) Invernizzi, M.; Piaggi, P. M.; Parrinello, M. Unified Approach to Enhanced Sampling. *Phys. Rev. X* **2020**, *10*, 041034, DOI: 10.1103/PhysRevX.10.041034.

(11) Tribello, G. A.; Bonomi, M.; Branduardi, D.; Camilloni, C.; Bussi, G. PLUMED 2: New Feathers for an Old Bird. *Computer Physics Communications* **2014**, *185*, 604–613, DOI: 10.1016/j.cpc.2013.09.018.
