# Supplementary figures and images for: Finite-Temperature Evolution of Frenkel Defects in Hybrid Perovskites: Healing and Lead-Methylammonium Antisite Pairs
Source: ACS Appl Mater Interfaces. 2026 Apr 9;18(15):22071–80. doi: 10.1021/acsami.6c02743 (PMC13107386; doi:10.1021/acsami.6c02743)

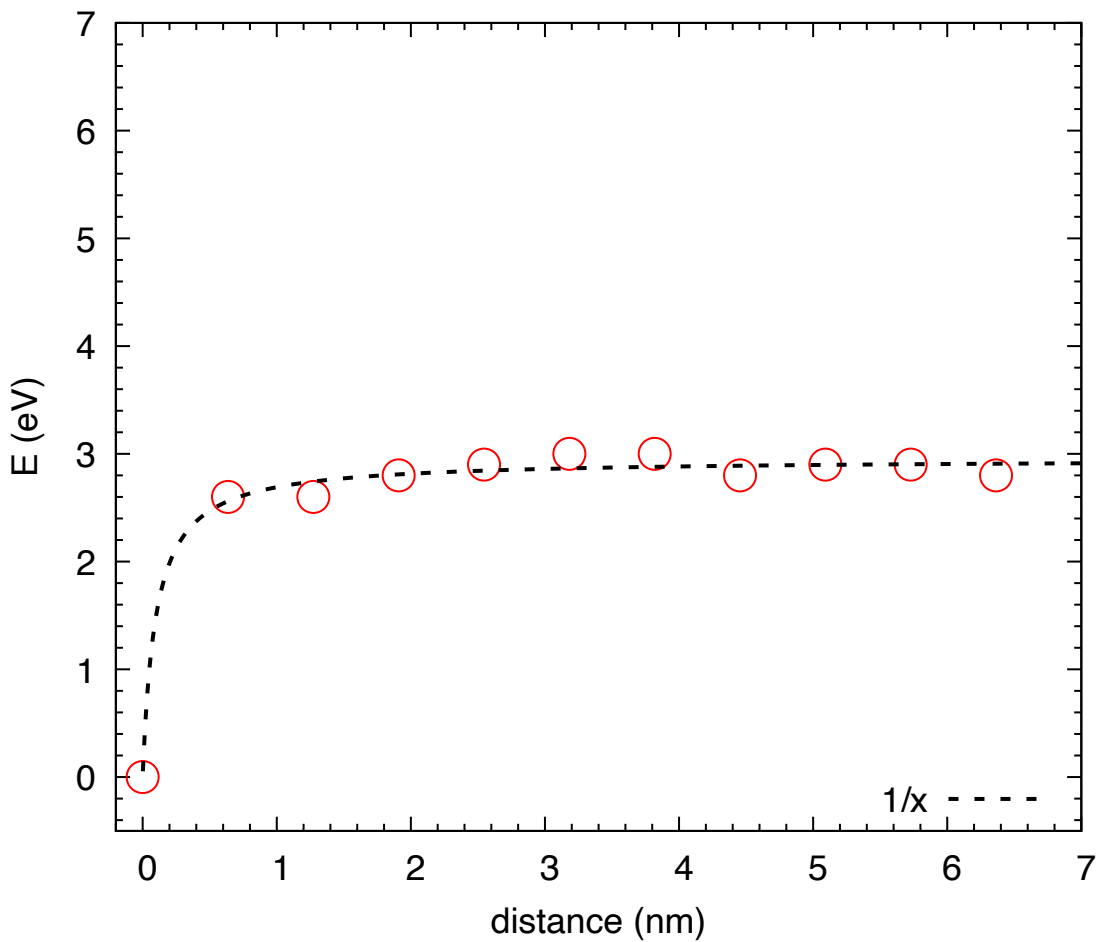

Supplement: Supplementary file 2 [file am6c02743_si_002.zip › supporting/fig4/PB_MA/basin.pdf]

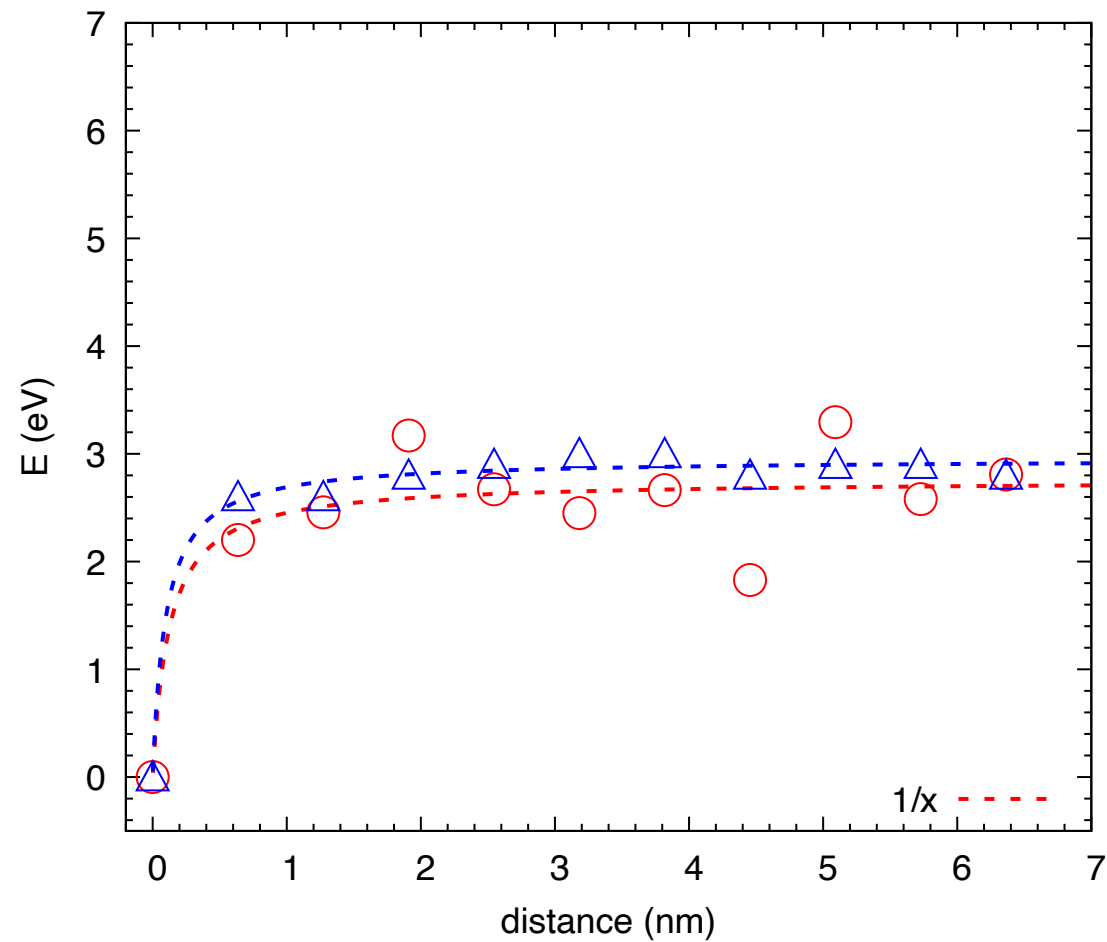

Supplement: Supplementary file 2 [file am6c02743_si_002.zip › supporting/fig4/PB_MA/basin300.pdf]

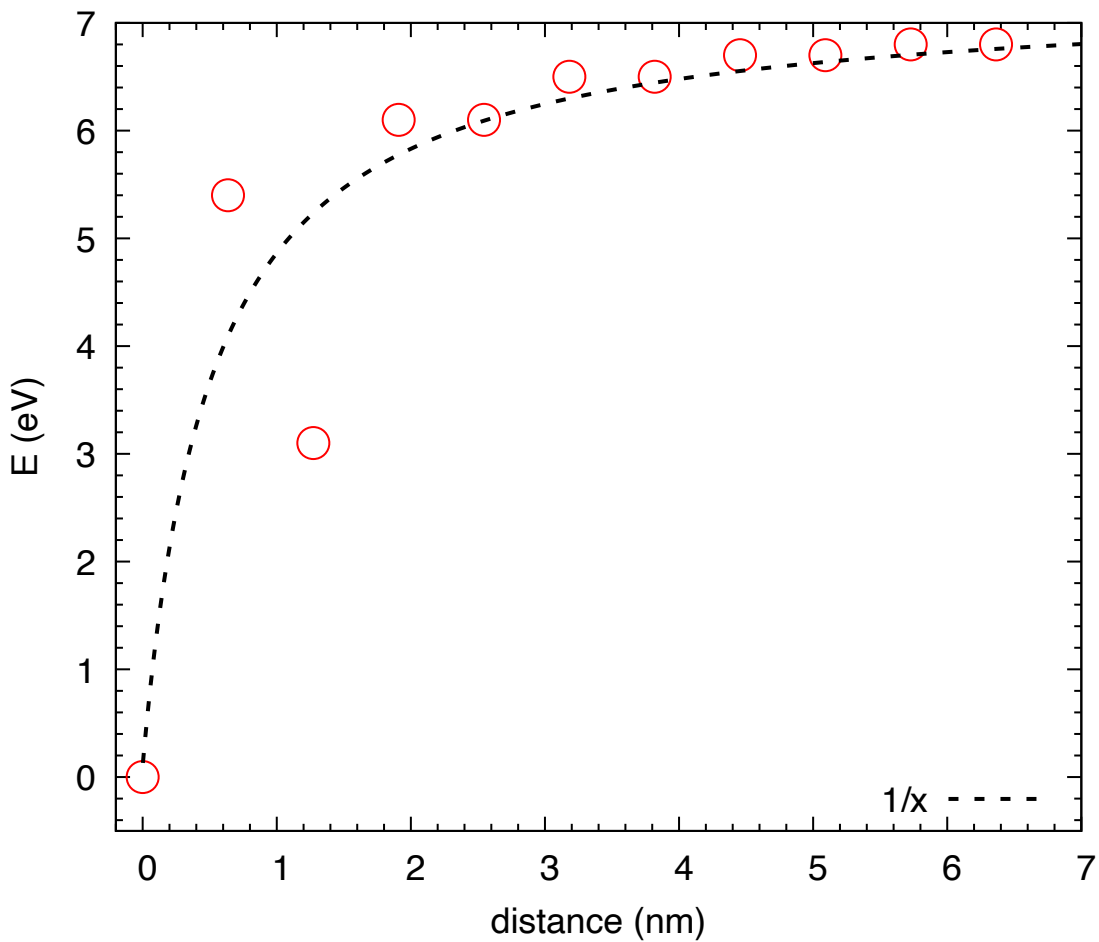

Supplement: Supplementary file 2 [file am6c02743_si_002.zip › supporting/fig4/PB/basin.pdf]

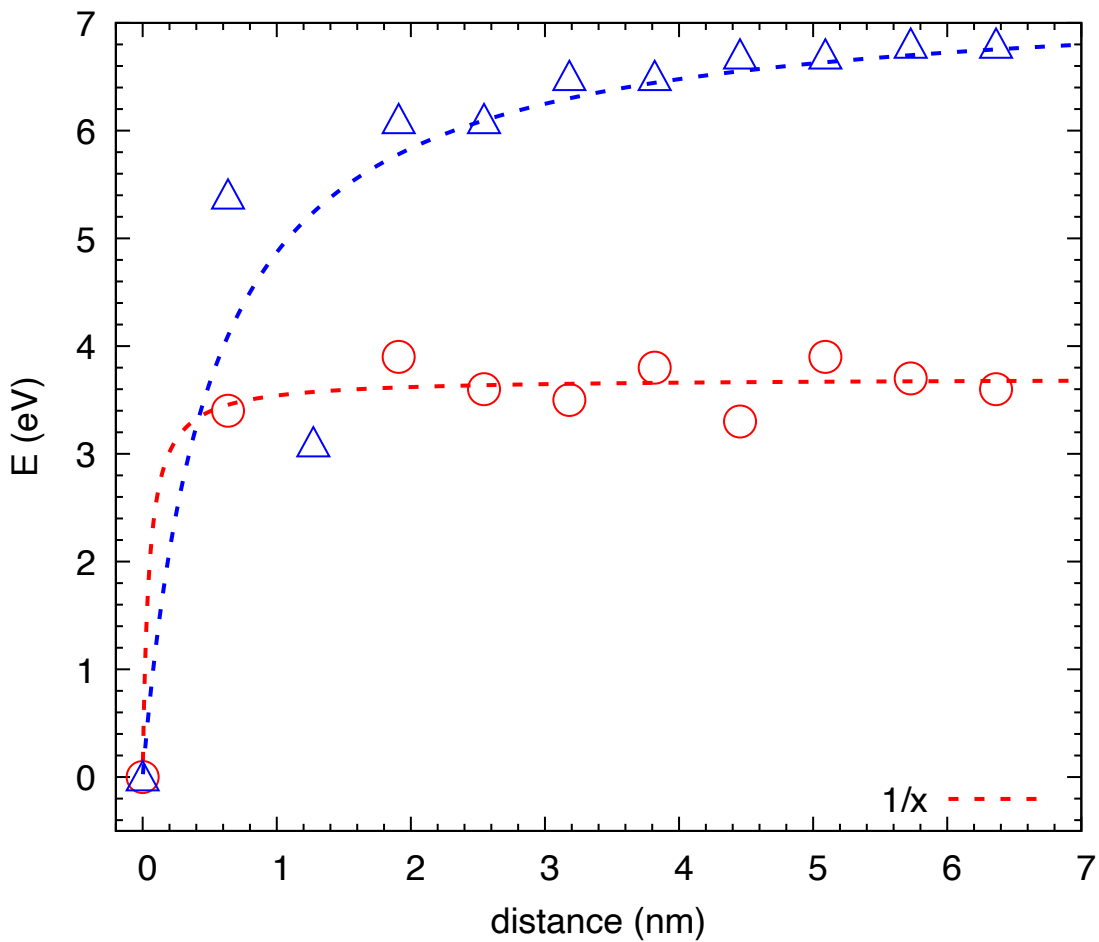

Supplement: Supplementary file 2 [file am6c02743_si_002.zip › supporting/fig4/PB/basin300.pdf]

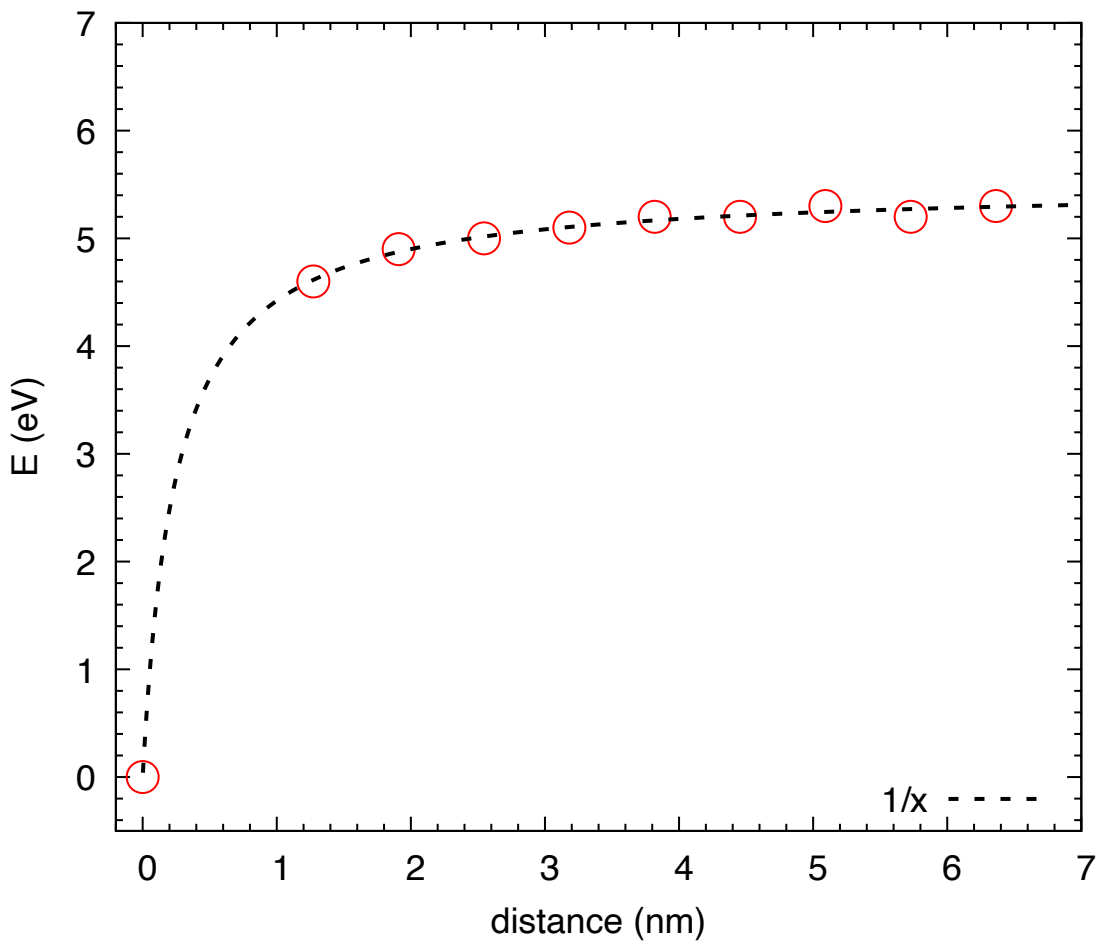

Supplement: Supplementary file 2 [file am6c02743_si_002.zip › supporting/fig4/MA/basin.pdf]

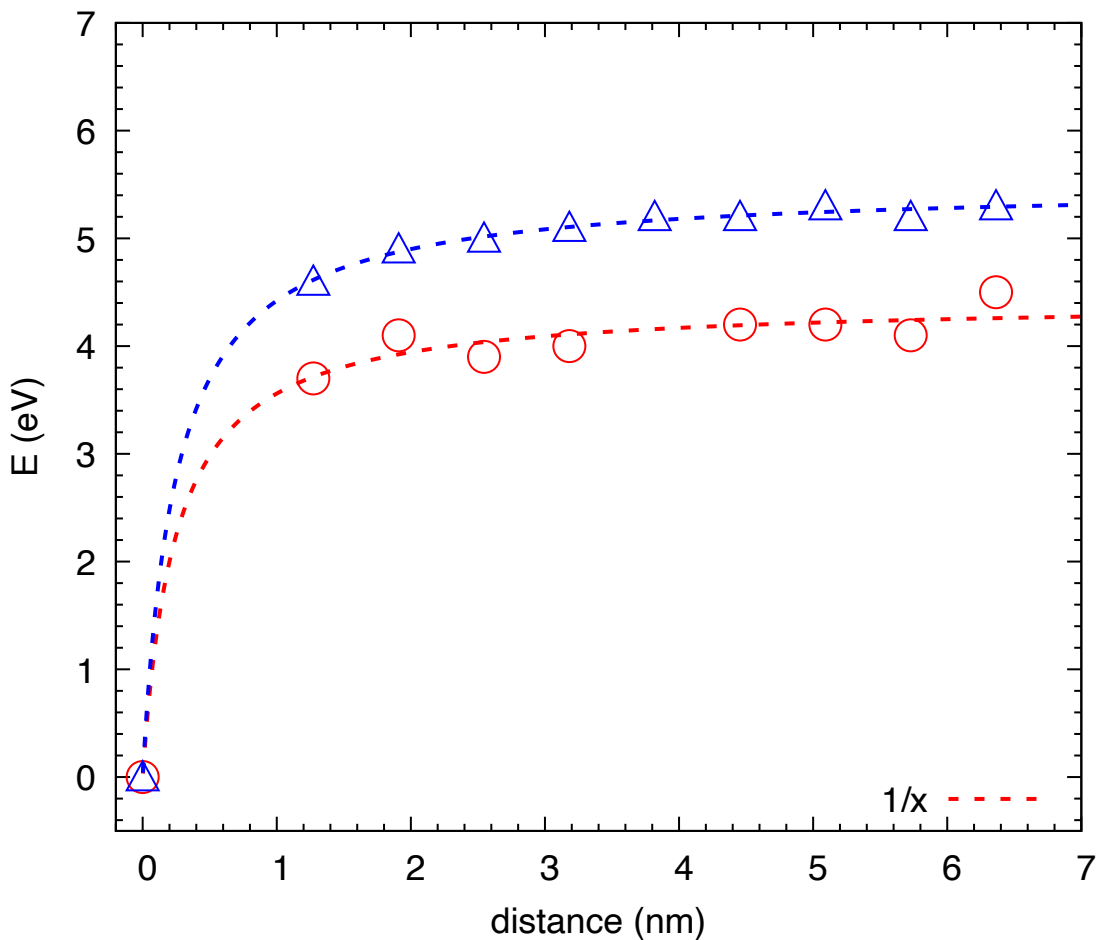

Supplement: Supplementary file 2 [file am6c02743_si_002.zip › supporting/fig4/MA/basin300.pdf]

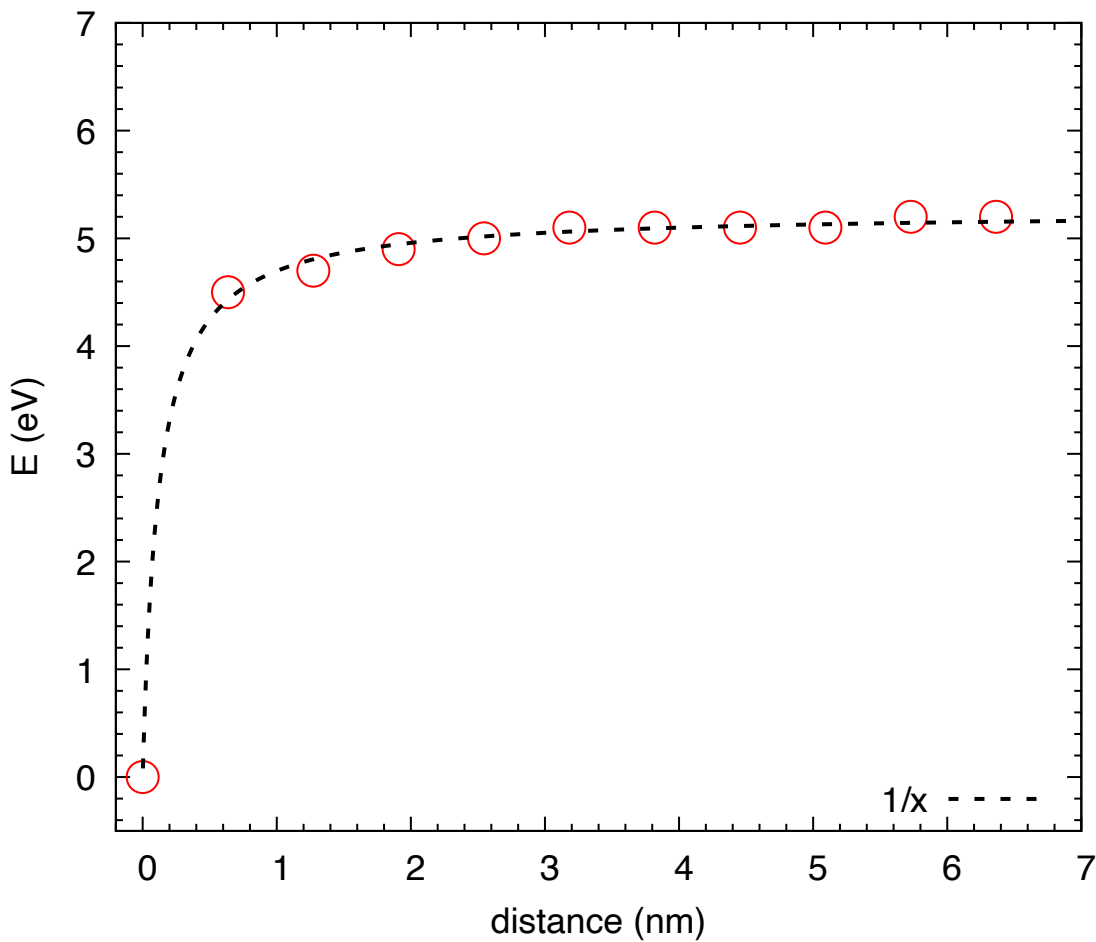

Supplement: Supplementary file 2 [file am6c02743_si_002.zip › supporting/fig4/I/basin.pdf]

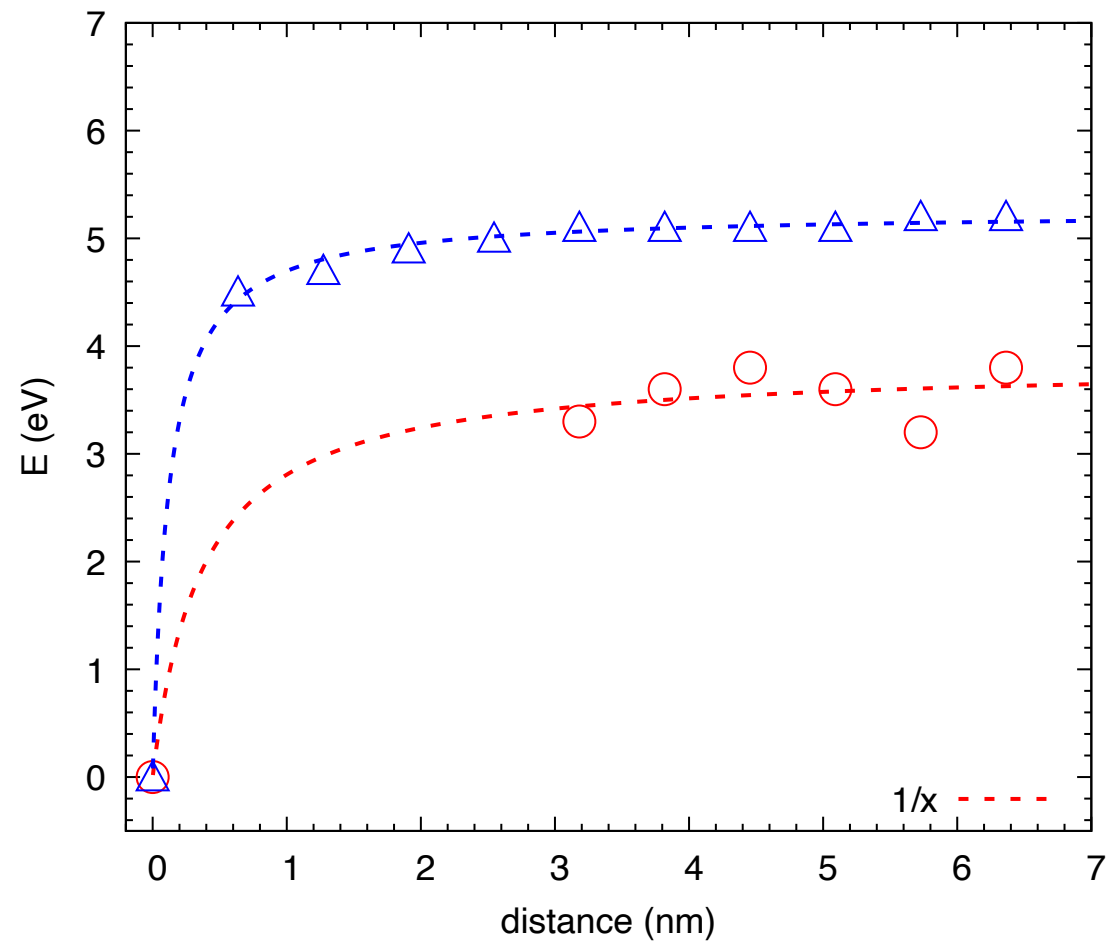

Supplement: Supplementary file 2 [file am6c02743_si_002.zip › supporting/fig4/I/basin300.pdf]
